# Amino acid **δ**^15^N values are associated with underweight status in a captive population of muskoxen

**DOI:** 10.1093/conphys/coag080

**Published:** 2026-10-22

**Authors:** Garrett A Savory, Molly Cook, Greg A Breed, John Blake, Diane M O’Brien

**Affiliations:** Institute of Arctic Biology, University of Alaska Fairbanks, 2140 Koyukuk Drive, Fairbanks, Alaska 99775-7000, USA; Department of Biology and Wildlife, University of Alaska Fairbanks, 2090 Koyukuk Drive, Fairbanks, Alaska 99775-6100 USA; Institute of Arctic Biology, University of Alaska Fairbanks, 2140 Koyukuk Drive, Fairbanks, Alaska 99775-7000, USA; Department of Biology and Wildlife, University of Alaska Fairbanks, 2090 Koyukuk Drive, Fairbanks, Alaska 99775-6100 USA; Institute of Arctic Biology, University of Alaska Fairbanks, 2140 Koyukuk Drive, Fairbanks, Alaska 99775-7000, USA; Department of Biology and Wildlife, University of Alaska Fairbanks, 2090 Koyukuk Drive, Fairbanks, Alaska 99775-6100 USA; Animal Resources Center, University of Alaska Fairbanks, 1033 Sheenjack Drive1033 Sheenjack Drive, Fairbanks, Alaska 99775-6980, USA; Institute of Arctic Biology, University of Alaska Fairbanks, 2140 Koyukuk Drive, Fairbanks, Alaska 99775-7000, USA; Department of Biology and Wildlife, University of Alaska Fairbanks, 2090 Koyukuk Drive, Fairbanks, Alaska 99775-6100 USA

**Keywords:** Body mass loss, compound specific, general linear mixed-effect regression, nitrogen stable isotope ratios, ungulate, wildlife health

## Abstract

Measures of protein balance, particularly changes in body mass, are important in understanding the health of wildlife populations but can be difficult or impossible to obtain from free-living animals. One potential biomarker, the δ^15^N value of bulk tissue, has had mixed associations with body mass. δ^15^N values of amino acids (AAs) have been shown to be more sensitive measures of trophic position than the bulk δ^15^N value and may also be more sensitive to changes in body mass. Here we evaluate AA δ^15^N values as a method to assess underweight status using archived serum samples from a captive population of muskoxen (*Ovibos moschatus*) that had decreased body mass resulting from an inadvertent dietary trace element deficiency. We predicted that AA δ^15^N values would be associated with deviations from the expected healthy body mass based on sex and age, and that they would be more sensitive than bulk δ^15^N values. We evaluated several linear mixed-effect models with δ^15^N values of bulk serum, AAs and other variables against deviations from expected body mass. In females, the δ^15^N values of glutamate/glutamine, leucine, aspartate/asparagine and serine, plus pregnancy status, were best associated with the difference from expected mass; in males, the δ^15^N values of glutamate/glutamine, leucine and proline were best associated with the difference from expected mass. Bulk δ^15^N values were not associated with the difference from expected mass. δ^15^N values of AAs have potential as a tool for researchers to evaluate body condition in animals.

## Introduction

Measures of protein balance, particularly changes in body condition, are critical in studies of wildlife health and nutrition. Nevertheless, accurate estimates of protein balance can be difficult to obtain, especially from free living animals (Barboza *et al.,* 2009). Nitrogen stable isotope analysis of bulk tissue and urine has been used to assess protein balance in animals (Gannes *et al.,* 1998; Kelly, 2000; Doi *et al.,* 2017), with higher δ^15^N values in body tissues associated with unreplaced protein mass loss (Gustine *et al.,* 2012; Lee *et al.,* 2012; Aguilar *et al.,* 2014; Cantalapiedra-Hijar *et al.,* 2015; Barboza *et al.,* 2020). Generally, the δ^15^N values of an animal’s diet are reflected in body tissues, with a tendency for body δ^15^N values to be ~3–4‰ higher relative to diet (DeNiro and Epstein, 1981; Macko *et al.,* 1982; Minagawa and Wada, 1984; Hobson and Welch, 1992; Post, 2002). This offset, termed trophic level enrichment, is caused by the disproportionately higher loss of ^14^N relative to ^15^N in waste nitrogen and is stable when diets are adequate and unchanging. However, when an animal fails to meet its dietary protein needs, the body δ^15^N value is expected to increase as the proportion of dietary nitrogen lost to waste increases (Koch, 2008). However, the association with protein loss can be inconsistent, with some studies finding no change in δ^15^N values despite significant loss of body mass (Ben-David *et al.,* 1999; Kempster *et al.,* 2007; Gómez-Campos *et al.,* 2011). Changes in δ^15^N values due to protein balance can be confounded with changes of δ^15^N values due to diet or movement (Finstad and Kielland, 2011; Gustine *et al.,* 2011a, 2011b, 2012; VanSomeren *et al.,* 2017; Barboza *et al.,* 2020) and may also be affected by tissue anabolic/catabolic state (Lee *et al.,* 2012). Assessing protein balance in animals using δ^15^N values requires more refined approaches to remove these confounding effects.

δ^15^N values of specific amino acids (AAs) have the potential to be a more sensitive biomarker of protein balance that could avoid the issue of confounding dietary and environmental effects. Starting in 2002 (McClelland and Montoya, 2002), numerous studies have reported variations in trophic level enrichment among AAs, especially at lower trophic levels (Chikaraishi *et al.,* 2009; Hoen *et al.,* 2014; McMahon *et al.,* 2015; Kendall *et al.,* 2017; Whiteman *et al.,* 2021). Several authors have noted that the AAs that show large enrichments in δ^15^N are those with amino groups that are highly interchangeable during protein metabolism, often termed ‘trophic’ AAs in food web studies (e.g. glutamate; Glu) (Petzke *et al.,* 2005; McMahon and McCarthy, 2016; O’Connell, 2017; Ishikawa, 2018). Non-interchangeable or ‘source’ AAs have little to no exchange of amine groups and reflect diet δ^15^N values with high fidelity (e.g. phenylalanine) (McMahon and McCarthy, 2016; O’Connell, 2017; Ishikawa, 2018). In food web studies, the difference in δ^15^N values between AAs with interchangeable (e.g. Glu) and non-interchangeable (e.g. phenylalanine; Phe) amines has been used to estimate trophic position, with AAs with non-interchangeable amines serving as the δ^15^N baseline (Chikaraishi *et al.,* 2009; Hoen *et al.,* 2014; McMahon *et al.,* 2015; Kendall *et al.,* 2017). Because underweight status due to protein loss can have similar effects on animal δ^15^N as increased trophic level, differences in δ^15^N values between AAs with interchangeable and non-interchangeable amines may also serve as a more sensitive and informative measure of protein balance (Petzke *et al.,* 2005; McMahon and McCarthy, 2016; O’Connell, 2017; Ishikawa, 2018). This may be particularly true for animals like herbivores for which trophic position is well known and constrained.

We examined δ^15^N values of AAs in archived blood serum (hereafter referred to as ‘serum’) samples collected from muskoxen (*Ovibos moschatus*) prior to, during and after a 2010 morbidity–mortality event at the University of Alaska Fairbanks, Robert G. White Large Animal Research Station (LARS). The morbidity–mortality event resulted from feed that was inadvertently marginal to deficient in trace minerals, notably copper and cobalt. This trace mineral deficiency caused lack of appetite and non-consumption of food, resulting in considerable weight loss and poor body condition in most animals. After this morbidity–mortality event, the diet was corrected to account for the trace mineral deficiency and the population recovered. This event provided an opportunity to evaluate associations between δ^15^N values of AAs and protein balance. To characterize the body mass status of the affected muskoxen, we generated expected body mass curves for muskoxen by sex and age, using data from healthy individuals after the morbidity–mortality event. We then modelled the deviation from expected healthy body mass through the event, using serum AA δ^15^N values, total serum (‘bulk’) δ^15^N values, and demographic variables. We predicted that the difference between δ^15^N values of AAs with interchangeable and non-interchangeable amines would be associated with underweight status, and that δ^15^N values of AAs would be more sensitive to underweight status than bulk δ^15^N values. Specifically, we predicted that the δ^15^N values of Glu (corrected for dietary effects with Phe) would likely increase with decreases from expected mass, for two reasons. First, Glu is the key hub for amine transfer in the generation of waste nitrogen (Brosnan, 2000), a process that is known to prefer ^14^N and is likely accelerated during mass loss as tissue protein is catabolized for energy. Second, numerous foodweb studies have consistently identified Glu as having one of, if not the most, elevated δ^15^N values with trophic level, which likely results from the same isotopic preference.

## Materials and methods

### Samples and design

To model the expected body mass of healthy muskoxen, we used animal management and veterinary medical records from LARS. We used 235 female and 147 male body mass measurements from 33 individual muskoxen between the ages of 4 months and 17 years to model expected body mass. All data used to model expected body mass came from records between 2012 and 2020, after muskoxen diets were corrected following the morbidity–mortality event. We did not use mass data from individuals prior to the morbidity–mortality event because health effects caused by the marginal to deficient dietary trace minerals likely developed over several preceding years. Therefore, weight data from 2010 and earlier were considered unreliable for modelling expected healthy body mass. Additionally, we excluded data points from individuals that were near death or pregnant, which would have also represented atypical body condition. Finally, we excluded one male and his offspring because he was significantly larger and originated from a different geographic region from other herd members.

To examine associations between δ^15^N values and body mass, we analysed 159 muskoxen serum samples archived by the University of Alaska Fairbanks, Animal Resources Center. Serum samples and body mass measurements were collected simultaneously from 34 individuals during regular preventive medicine and clinical case examinations from 2007 to 2016. The turnover rate of stable isotopes in blood serum has not been estimated for muskoxen. Estimates from studies of other medium- to large-bodied mammals suggest that muskoxen serum should turn over in approximately 1 to 3 months (Caut *et al.,* 2011; Bahar *et al.,* 2014; Johnson *et al.,* 2021). We used serum samples from individuals at least 3 years old. We also collected information on date of sampling and individual age. Additionally, we collected pregnancy and lactation status in females, and whether males were intact bulls or castrates. All LARS muskoxen have been maintained under the University of Alaska Fairbanks, Institutional Animal Care and Use Committee (IACUC) husbandry protocols in addition to IACUC research protocols for active projects. Because no new samples were collected for the purposes of this study, it did not require an IACUC research protocol.

## Laboratory methods

### Total serum or ‘bulk’ nitrogen isotope ratios

For bulk δ^15^N analysis, we used 3.5 μl of serum. Serum was pipetted directly into tin capsules and dried at 60°C for 24 h. Samples were analysed at the Alaska Stable Isotope Facility using continuous-flow isotope ratio mass spectrometry with Costech ECS4010 Elemental Analyser (Costech Analytical Technologies Inc., Valencia, CA, USA) interfaced to a Finnigan Delta Plus XP isotope ratio mass spectrometer via the Conflo III interface (Thermo Fisher Scientific, Waltham, MA, USA). δ^15^N values are nitrogen isotope ratios expressed in δ notation as per mil (per thousand, ‰) relative abundance of heavy isotope referenced to a standard, atmospheric N_2_ (AN) (Eq. 1). Laboratory standards were measured for every 10th sample to assess precision and accuracy. Standard reference materials (peptone) of bulk δ^15^N measurements (expected value = +7.0‰) were +6.6 to +7.3‰ (SD ≤ 0.3‰) within runs and a mean +7.0 ± 0.1‰ among runs. We additionally checked all sample batches for run order effects.


(1) \begin{equation*} \mathrm{\delta} {}^{15}\mathrm{N}=\frac{\left(\frac{{}^{15}{\mathrm{N}}_{\mathrm{sample}}}{{}^{14}{\mathrm{N}}_{\mathrm{sample}}}\right)}{\left(\frac{{}^{15}{\mathrm{N}}_{\mathrm{AN}}}{{}^{14}{\mathrm{N}}_{\mathrm{AN}}}\right)}-1 \end{equation*}

### A‌A nitrogen isotope ratios

To measure the δ^15^N values of individual AAs, we used 24 μl of serum. We released AAs from proteins by hydrolysing in 6 N HCl at 110°C for 20 h, after flushing vials with N_2_. We extracted lipids with n-hexane and dichloromethane (6:5 v/v) and evaporated samples to dryness by heating at 70°C under a stream of N_2_. We derivatized AA samples and standards to N-acetyl methyl esters using methods developed by Corr *et al.* (2007) and Larsen *et al.* (2013). Briefly, the process involves a methylation reaction with acidified methanol (1 h at 75°C) followed by an acetylation reaction with acetic anhydride, triethylamine and acetone (10 min at 60°C). We flushed tubes with N_2_ prior to heating and dried between steps under N_2_. We purified AA derivatives using two rounds of phosphate buffer washes, extracted AAs in chloroform and then evaporated samples to dryness under N_2_ (McMahon *et al.,* 2018). We dissolved derivatized AAs in ethyl acetate and stored at −20°C.

AAs were separated and δ^15^N values were measured with gas chromatography–combustion–isotope ratio mass spectrometry. We analysed samples in triplicate using a GC-IsoLink System interfaced with a Delta V isotope ratio mass spectrometer (Thermo Fisher Scientific). We obtained reliable δ^15^N values from eight AA peaks: aspartate/asparagine (Asx), Glu/glutamine (Glx), leucine (Leu), Phe, proline (Pro), serine (Ser), threonine (Thr) and valine (Val). During hydrolysis, glutamine and asparagine are deamidated and converted into Glu and aspartate (Silverman *et al.,* 2022); a single analyte is measured for each, expressed as Glx and Asx. We checked results for quality assurance and control using internal checks and standards.

### Analytical batch design and AA δ^15^N standards

To avoid the possibility of confounding changes due to body mass with batch effects, all samples from a single individual were measured in the same batch, or two (split evenly) if the number of samples was larger than could be derivatized in a single batch. Samples from each derivative batch were randomly assigned a run order. Due to instrument limitations on the number of samples that could be analysed in a single run, derivative batches were split into two or three runs (depending on the size of the derivative batch).

We adjusted the measured AA δ^15^N values using a derivatized mixed AA standard (AA_1_) that included all the AAs measured in this study, with known δ^15^N values (Supplemental Information 1). AA_1_ was included in every batch and measured in triplicate with injections at the beginning, middle and end of a run. We used a second derivatized mixed AA standard (AA_2_) with known different δ^15^N values to monitor and correct for run order effects. AA_2_ was measured five times and interspersed evenly from beginning to end of a batch run. To test for run order effects within batches, we regressed AA_2_ δ^15^N values on run order for each AA. We applied a linear correction for run order if the slope was ≥0.005 and *R*^2^ was ≥0.5, which corrected subsequent injections relative to the first usable AA_2_ injection. Across all batches (*n* = 28), 8 batches of Asx, 1 batch of Glx, 7 batches of Leu, 10 batches of Phe, 11 batches of Pro, 10 batches of Ser, 3 batches of Thr and 7 batches of Val were corrected for run order effects. Two-sided *F*-tests for paired samples showed no significant differences (*α* level = 0.05) in the variances when comparing non-corrected AA_2_ δ^15^N values for run order and corrected AA_2_ δ^15^N values for run order: Asx (1.0 and 0.8, *F*_1, 129_ = 1.3, *P* = 0.19), Glx (0.2 and 0.2, *F*_1, 129_ = 1.0, *P* = 0.85), Leu (1.3 and 1.4, *F*_1, 106_ = 0.9, *P* = 0.67), Phe (0.1 and 0.1, *F*_1, 130_ = 0.8, *P* = 0.13), Pro (0.9 and 0.9, *F*_1, 130_ = 1.0, *P* = 0.85), Ser (0.8 and 0.9, *F*_1, 129_ = 0.9, *P* = 0.50), Thr (0.6 and 0.6, *F*_1, 129_ = 1.1, *P* = 0.63) and Val (0.3 and 0.4, *F*_1, 129_ = 0.8, *P* = 0.11). To scale normalize the data, we subtracted the difference between the run order corrected AA_1_ within each batch and the known AA_1_ values from all of our AA δ^15^N data (Yarnes and Herszage, 2017).

A derivatized ‘check sample’ of horse (*Equus ferus caballus*) serum was used to evaluate error across batches. Check samples were placed randomly, like a sample, in every derivative batch and were analysed in triplicate. A check sample from every derivatized batch was measured but was not in every instrument run for a batch, as these were smaller than the derivatization batches. Furthermore, we added two co-injected ‘internal’ standards to all samples and mixed AA standards to monitor instrument performance across all injections, one derivatized (norleucine; two different values used in different batches) and one non-derivatized (caffeine). Mean check sample AA δ^15^N values across multiple injections within a batch (with maximum SD reported) were Asx +10.2 to +12.3‰ (SD ≤ 0.8), Glx = +11.8 to +13.3‰ (SD ≤ 0.9), Leu = +9.2 to +12.9‰ (SD ≤ 0.8), Phe = +10.6 to +12.5‰ (SD ≤ 1.0), Pro = +13.3 to +19.2‰ (SD ≤ 0.9), Ser = +7.7 to +10.5‰ (SD ≤ 1.2), Thr = −2.1 to 0.0‰ (SD ≤ 0.8) and Val = +9.8 to +14.0‰ (SD ≤ 2.0). The mean ± SD of δ^15^N values of AAs of the serum check samples across all batches were Asx = +10.9 ± 1.2‰, Glx = +12.7 ± 0.5‰, Leu = +11.5 ± 1.4‰, Phe = +11.7 ± 0.8‰, Pro = +17.9 ± 1.4‰, Ser = +9.1 ± 0.8‰, Thr = −0.9 ± 0.8‰ and Val = +12.2 ± 1.3‰. The mean δ^15^N values of norleucine across multiple injections within batches (with maximum within batch SD reported) were +9.4 to +10.4‰ (SD ≤ 0.5) for norleucine #1 and +17.2 to +21.3‰ (SD ≤ 2.5) for norleucine #2. The mean ± SD of δ^15^N values of norleucine of the serum check samples across batches were +9.8 ± 0.5‰ for norleucine #1 and +19.9 ± 1.4‰ for norleucine #2. The range of mean δ^15^N values of the serum check samples of caffeine across multiple injections within batches (with maximum within batch SD reported) was −1.8 to −3.3‰ (SD ≤ 0.5) and the mean ± SD of δ^15^N values of caffeine across batches was −2.3 ± 0.4‰.

## Data analysis

### Growth models

We modelled the body mass of healthy muskoxen as a function of age (in days) and sex using nonlinear regression. We evaluated six non-linear models: two-parameter asymptotic exponential, two-parameter asymptotic exponential through the origin, two-parameter logistic, Weibull, Gompertz and Michaelis–Menten. We used R packages *nlme* (Pinheiro *et al.,* 2024) and *nlstools* (Baty *et al.,* 2015) to fit self-starting models for each sex. We checked model residuals for normality with a Shapiro–Wilk test and autocorrelation with a runs test. From the set of models that passed both normality and autocorrelation tests, we used AICc (Akaike information criteria corrected for small sample size) to find the best approximating model in R package *MuMIn* (Bartoń, 2024). We calculated pseudo-*R*^2^ to assess the fits of best approximating models in R package *statforbiology* (Onofri, 2025) and calculated residual standard error to assess precision of predictions. We used the best model for each sex to estimate the expected healthy mass of individuals during the morbidity–mortality event, based on individual sex and age.

### Underweight status models

To evaluate whether AA stable nitrogen isotope ratios could be used to characterize underweight status, we modelled muskoxen body condition on stable isotope data for females and males using general linear mixed-effect regression using the R package *lme4* (Bates *et al.,* 2015). To characterize body condition, we used the difference between measured and expected body mass (Diff_EM_) based on the growth models described above. We assessed four sets of models in a hierarchical approach using AICc to build our final models for female and male muskoxen. The equation for the full model was Eq. (2), where *β*_0_ is the intercept, *β_x_* is a vector of the fixed effects with non-δ^15^N variables (season, pregnancy status, castrated status), *α_x_* is a vector of the fixed effects with δ^15^N variables (bulk, Asx, Glx, Leu, Pro, Ser, Thr, Val), *θ_x_* is a vector of the random effects (*Animal ID*, year) and ε is the residual error.


(2) \begin{equation*} Y\left({\mathrm{Diff}}_{\mathrm{EM}}\right)={\beta}_0+{\beta}_x+{\alpha}_x:{\beta}_x+{\theta}_x+\varepsilon \end{equation*}

Tissue δ^15^N values reflect changes in an animal’s diet in addition to metabolic effects. We used the δ^15^N value of Phe to account for dietary effects on study δ^15^N values (both AAs and bulk serum) because it undergoes little, if any, trans-amination and does not appear to have metabolic effects (McMahon and McCarthy, 2016; O’Connell, 2017). In preliminary analyses (Supplemental Information 2), we found that Phe δ^15^N values were not associated with Diff_EM_ but were associated with year and season in both female and male muskoxen (Fig. 1). The δ^15^N values of Phe ranged by ~5.0‰, which likely represents changes in diet over different time periods and/or individual dietary preferences (e.g. different commercial feeds and hay sources, feeding on greater proportion of pasture grass in the summer and autumn versus feeding on a greater proportion of commercial feed and hay in winter). We used Eqs (3) and (4) to correct for changes in dietary δ^15^N in our analyses. Offsetting AA δ^15^N values with Phe did not significantly change the variance of any of the AA δ^15^N values (Supplemental Information 2).

**Figure 1:**
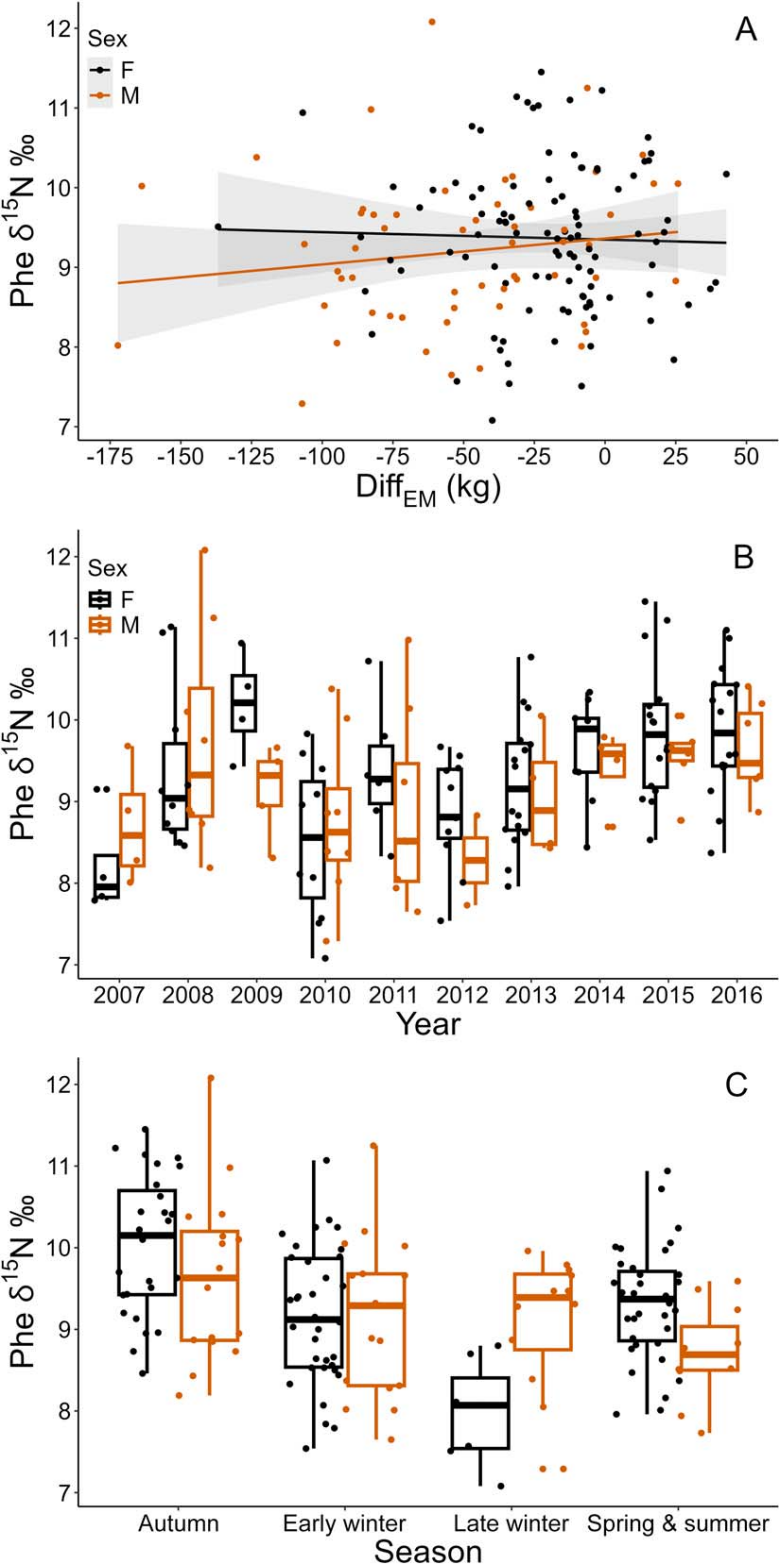
Phe δ^15^N values of female and male muskoxen serum by difference from expected mass (Diff_EM_) (**A**), Year (**B**) and Season (**C**). In A, shaded areas represent the standard error band. In B and C, the middle horizontal lines represent the mean, the boxes represent 25% and 75% quantiles and the vertical lines represent the 95% quantile.


(3) \begin{equation*} {\mathrm{AA}}_{\mathrm{D}}=\mathrm{AA}\ \mathrm{\delta} {}^{15}\mathrm{N}-\mathrm{Phe}\ \mathrm{\delta} {}^{15}\mathrm{N} \end{equation*}

(4) \begin{equation*} {\mathrm{bulk}}_{\mathrm{D}}=\mathrm{bulk}\ \mathrm{\delta} {}^{15}\mathrm{N}-\mathrm{Phe}\ \mathrm{\delta} {}^{15}\mathrm{N} \end{equation*}

In Model Set 1, we evaluated two additive models for each sex with random effects, *Animal ID* and *Animal ID* + *Year*. *Animal ID* represented unique individuals, and *Year* was a categorical variable for each year of the study (2007–2016). All random effect models included *Animal ID* as a variable to prevent pseudo-replication. To assess random effect components in Model Set 1, we used restricted maximum likelihood to fit models and assess model selection.

In Model Set 2, we evaluated four mixed effect models for each sex, including additive fixed effects with non-δ^15^N variables that potentially affected muskoxen Diff_EM_, and including the random effect variables selected from the best approximating model from Model Set 1. Models of both sexes included a categorical variable *Season* that included four seasons based on potential changes in muskoxen mass through the year given known seasonal differences in their life history: Spring and Summer (May–July), Autumn (August–September), Early Winter (October–January) and Late Winter (February–April) (Barboza *et al.,* 2006). Pregnant females tend to have more mass than non-pregnant individuals; for female models, we included *Pregnant* as a variable. Castrated males do not go through a rut during breeding season and, as a result, avoid the rut-related weight loss experienced by intact bulls; for male models, we included *Castrated* as a variable. We also included a model with only random effects to assess as a null model. For model selection in Model Set 2, and all subsequent fixed effects models, we used full maximum likelihood to fit models and assess model selection.

In Model Set 3, we evaluated 107 mixed effect models for each sex using fixed additive effects from multiple combinations of the seven AA_D_s, a model with only bulk_D_ and a model with only bulk; the additive fixed effect variables from the best approximating models from Model Set 2; and the random effect variables from Model Set 1. We included a model with only random effects and fixed effects of non-δ^15^N variables to assess as a null model.

In Model Set 4, we evaluated interactive effects of fixed effects with variables from the best models from Model Sets 2 and 3. We included multiple two-way interactions between non-δ^15^N and δ^15^N values (e.g. Leu_D_ + Glx_D_ + *Pregnant*: Leu_D_ + *Pregnant*: Glx_D_). Models in Set 4 included the random effects and additive fixed effects carried over from Model Set 3. We included the best model from Model Set 3 to assess as a null model.

We checked models for normality and homoscedasticity by visual examination of quantile–quantile plots and plotting model fits against model residuals. To estimate prediction error, we bootstrapped root-mean-square errors for the best approximating models using R package *insurancerating* (Haringa, 2024).

Additionally, we evaluated how multiple variables, including Diff_EM_, affected bulk and AA δ^15^N values. Details on data analysis and results are in Supplemental Information 3 and 4.

## Results

For the muskoxen growth curve models, we found that the two-parameter asymptotic exponential through the origin model (Eq. (5)) was the best approximating model for females (Table 1 and Fig. 2) and that the Gompertz model (Eq. (6)) was the best approximating model for males (Table 1 and Fig. 2). Predicted mean mass of fully grown muskoxen was 239.9 kg [234.2–243.6, 95% confidence interval (CI)] for females and 312.3 kg (294.4–330.2, 95% CI) for males (Table 2). The female model had a pseudo-*R*^2^ = 0.83 and a residual standard error = 22.1 kg. The male model had a pseudo-*R*^2^ = 0.83 and had a residual standard error = 32.7 kg. In the population that we evaluated for body condition during the morbidity–mortality event, the mean Diff_EM_ was −23.9 kg (SD = 31.6) for non-pregnant females, −7.3 kg (SD = 21.5) for pregnant females and −51.2 kg (SD = 43.1) for males. Diff_EM_ ranged from a low of −172.3 kg (a 3-year-old male that was euthanized during the morbidity–mortality event) to a high of 42.9 kg (a 4-year-old pregnant female).

**Table 1:** Model selection of non-linear growth models for female and male muskoxen from the University of Alaska Fairbanks, LARS

Female growth models	*K*	Δ AIC	*W*
Two-parameter asymptotic exponential (origin)	3	0.0	0.44
Two-parameter asymptotic exponential	4	1.0	0.27
Gompertz	4	2.0	0.16
Weibull	5	3.0	0.10
Two-parameter logistic	4	5.9	0.02
Michaelis–Menten	3	27.9	0.00
Male growth models	*K*	Δ AIC	*W*
Gompertz	4	0.0	0.40
Two-parameter logistic	4	0.5	0.32
Weibull	5	1.8	0.17
Two-parameter asymptotic exponential	4	2.7	0.11

*K* is the number of model parameters, Δ AIC is the difference of AIC values from the best approximating model and *W* is the AIC model weight.

**Figure 2:**
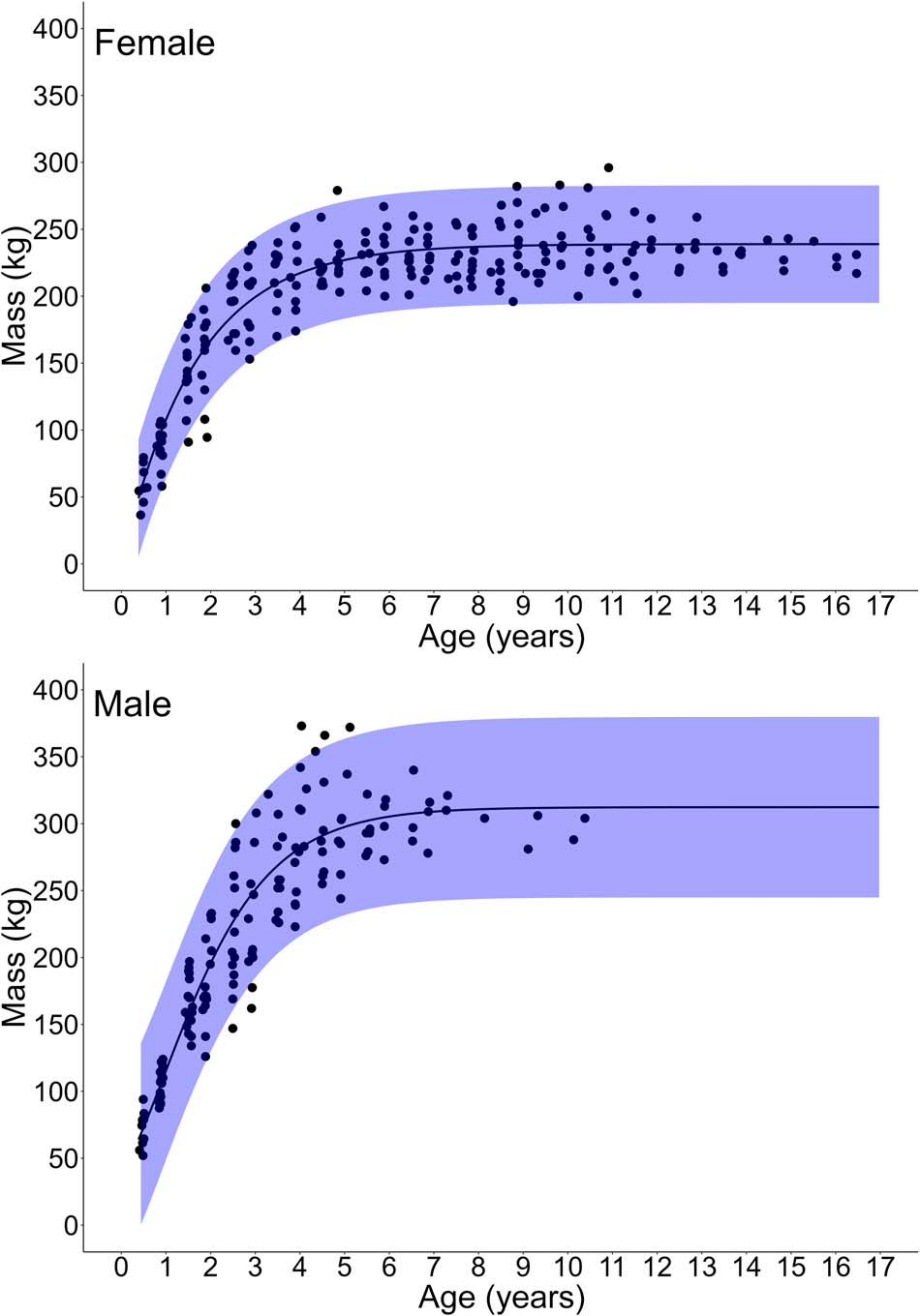
Modelled growth curves of female and male muskoxen from measurements taken from captive animals during routine health checks (2012–2020) at the University of Alaska Fairbanks, LARS. The lines represent the mean estimate, and the shaded regions represent 95% CIs. The prediction of female mass is from a two-parameter asymptotic exponential (origin) model, and the prediction male mass is from a Gompertz model. For this figure, we converted ‘age’ from days (as was used in the models) to years for ease of interpretation.


(5) \begin{equation*} \mathrm{mass}\ \left(\mathrm{kg}\right)=238.905\left(1-{e}^{\left(6.412\ast \mathrm{age}\right)}\right) \end{equation*}

(6) \begin{equation*} \mathrm{mass}\ \left(\mathrm{kg}\right)=312.300\ast{e}^{-2.117\ast{\mathrm{e}}^{-0.998\ast \mathrm{age}}} \end{equation*}

For female muskoxen, we found the best approximating model of Diff_EM_ included fixed additive effects of Asx_D_, Glx_D_, Leu_D_, Ser_D_ and *Pregnant* with random effects of *Animal ID* and *Year* on the intercept (Table 3, Supplemental Information 4). Estimated marginal pseudo-*R*^2^ was 0.309 and conditional pseudo-*R*^2^ was 0.816. Random effects account for 73.4% of the error (*Animal ID* = 61.9%, *Year* = 11.5%). Model predictive accuracy, estimated with mean bootstrapped root mean square error, was 11.0 kg (95% CI = 7.1, 14.9; bias = −1.9). Pregnancy status (*Pregnant*, *β* = 32.282; 95% CI = 23.391, 40.929) had the strongest association with Diff_EM_ in female muskoxen followed by Glx_D_ (*β* = −18.552, 95% CI = −27.834, −9.413), Ser_D_ (*β* = 11.132, 95% CI = 6.052, 16.354), Asx_D_ (*β* = 9.771; 95% CI = 3.688, 15.789) and Leu_D_ (*β* = −7.851; 95% CI = −12.304, −3.392) (Table 4 and Fig. 3). When non-pregnant female muskoxen had Glx_D_ δ^15^N values approximately above −1‰ (holding other AA δ^15^N values at their mean), they were predicted to be underweight. Pregnant female muskoxen were predicted to be underweight when their Glx_D_ δ^15^N values were above 0‰. None of the pregnant female muskoxen had Glx_D_ δ^15^N values above +1‰.

When evaluating effects on male muskoxen Diff_EM_ as a response, we found the best approximating model included fixed additive effects of Glx_D_, Leu_D_ and Pro_D_ with random effects of *Animal ID* and *Year* on the intercept (Table 5, Supplemental Information 5). Estimated marginal pseudo-*R*^2^ was 0.238 and conditional pseudo-*R*^2^ was 0.617. Random effects account for 49.8% of the error (*Animal ID* = 10.1%, *Year* = 39.7%). Model predictive accuracy, estimated with mean bootstrapped root mean square error, was 18.6 kg (95% CI = 7.6, 27.0; bias = −3.2). Glx_D_ (*β* = −16.596; 95% CI = −30.058, −3.262) had the strongest association with Diff_EM_ in male muskoxen, followed by Pro_D_ (*β* = 14.156; 95% CI = 2.695, 25.760) and Leu_D_ (*β* = −10.545; 95% CI = −17.170, −3.867) (Table 6 and Fig. 4). When male muskoxen had Glx_D_ δ^15^N values approximately above −1‰ (holding other AA δ^15^N values at their mean), they were predicted to be underweight.

**Table 2:** Estimates for non-linear growth curve models for female and male muskoxen from the University of Alaska Fairbanks, LARS

Female two-parameter asymptotic exponential (origin) model				
Coefficients	Estimates	95% CI	*t*-value	*P*-value
a	238.905	(234.175, 243.635)	99.7	<0.0001
b	−6.412	(−6.501, −6.323)	−142.5	<0.0001
Residual standard error = 22.1				
Male Gompertz model				
Coefficients	Estimates	95% CI	*t*-value	*P*-value
a	312.300	(294.444, 330.184)	34.8	<0.0001
b	2.117	(1.626, 2.609)	8.6	<0.0002
c	0.998	(0.997, 0.998)	4177.2	<0.0003
Residual standard error = 32.7				

**Table 3:** Model selection of female muskoxen with Diff_EM_ (the difference from expected mass, kg) as the response

Female Model Set 1 (random effects)	*K*	Δ AICc	*W*
*Animal ID + Year*	4	0.0	0.82
*Animal ID*	3	3.0	0.18
Female Model Set 2 (all include *Animal ID* + *Year*)	*K*	Δ AICc	*W*
*Pregnant*	5	0.0	0.91
*Pregnant* + *Season*	8	4.7	0.09
*Season*	7	17.8	0.00
Null	4	23.1	0.00
Female Model Set 3 (all include *Animal ID* + *Year*)	*K*	Δ AICc	*W*
*Pregnant + Asx_D_ + Glx_D_ + Leu_D_ + Ser_D_*	9	0.0	0.39
*Pregnant + Asx_D_ + Glx_D_ + Leu_D_ + Ser_D_ + Pro_D_*	10	1.3	0.20
*Pregnant*	5	23.5	0.00
*Pregnant + Bulk*	6	25.7	0.00
*Pregnant + Bulk_D_*	6	25.7	0.00
Female Model Set 4 (all include *Animal ID + Year*)	*K*	Δ AICc	*W*
*Pregnant + Asx_D_ + Glx_D_ + Leu_D_ + Ser_D_*	9	0.0	0.27
*Pregnant + Asx_D_ + Glx_D_ + Leu_D_ + Ser_D_ + Pregnant: Asx_D_*	10	1.5	0.13

*K* is the number of parameters in the model, Δ AIC is the difference of AIC values from the best approximating model and *W* is the AICc model weight. The subscript ‘D’ indicates that the δ^15^N value has been corrected for dietary (D) effects by subtraction of the Phe δ^15^N value. See Supplementary Table S8 for tables of full model selection results.

## Discussion

Our goal was to evaluate AA δ^15^N values as a method to assess underweight status in captive muskoxen. We first established an expected healthy mass by sex and age by modelling mass data from muskoxen in good body condition that were fed a complete and balanced diet. We then used these models to estimate Diff_EM_ (the difference from expected mass, kg) as a measure of underweight status in muskoxen during the morbidity–mortality event. The δ^15^N values of Phe did not vary with Diff_EM_ and were used to control for the effect of varying diet on AAs δ^15^N values. We evaluated several models of Diff_EM_ in female and male muskoxen using Phe-corrected AA δ^15^N values and compared these to models using the bulk δ^15^N value. We found that Phe-corrected values of Glx and Leu best predicted Diff_EM_ in both females and males, Asx and Ser only in females, and Pro only in males. We found that bulk δ^15^N values were not predictive of Diff_EM_, even when we accounted for dietary effects using δ^15^N Phe. Our best models were able to predict Diff_EM_ with relatively high accuracy.

**Table 4:** Model parameter estimates for prediction of female muskoxen Diff_EM_ (the difference from expected mass, kg)

Fixed effects	Estimate	95% CI	*t*-value
Intercept	−13.84	−29.92, 1.66	−1.72
*Pregnant*	32.28	23.39, 40.93	7.21
*Asx_D_*	9.77	3.69, 15.79	3.13
*Glx_D_*	−18.55	−27.83, −9.41	−3.90
*Leu_D_*	−7.85	−12.30, −3.39	−3.48
*Ser_D_*	11.13	6.05, 16.35	4.25
Random effects	SD	ICC	
*Animal ID*	23.2	0.619	
*Year*	10.0	0.115	
Residual	15.2		

Marginal pseudo-*R*^2^ = 0.309; conditional pseudo-*R*^2^ = 0.816.

The subscript ‘D’ indicates that the δ^15^N value has been corrected for dietary (D) effects by subtraction of the Phe δ^15^N value. ICC is intraclass correlation.

**Figure 3:**
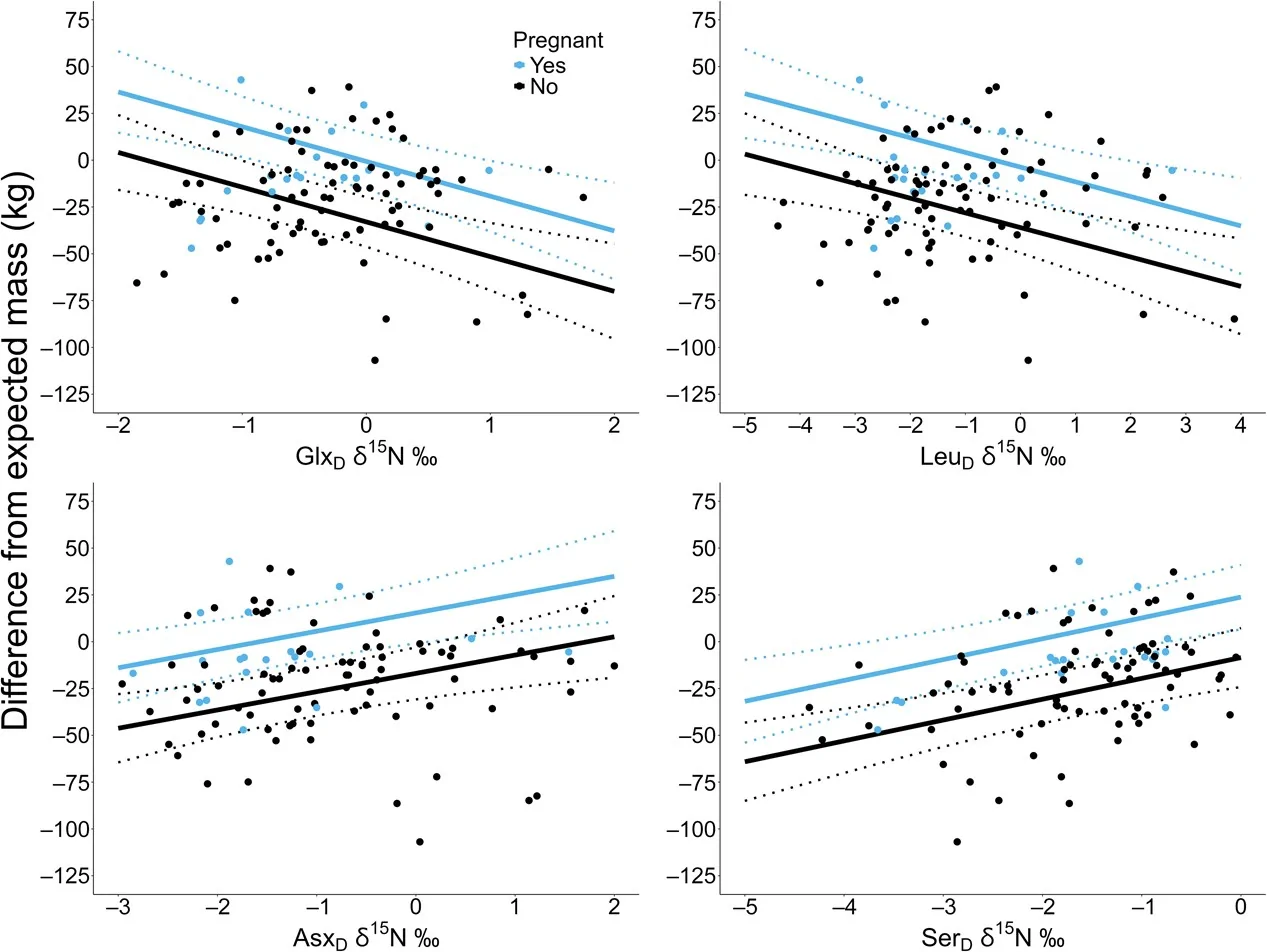
Predicted Diff_EM_ (the difference from expected mass, kg) for female muskoxen given their AA_D_ δ^15^N value. Dotted lines represent 95% CIs from model predictions. For each specific AA_D_ δ^15^N value, the other AA_D_ δ^15^N values were held at their mean. Points represent individual data points.

**Table 5:** Model selection of male muskoxen with Diff_EM_ (the difference from expected mass, kg) as the response

Male Model Set 1 (random effects)	*K*	Δ AICc	*W*
*Animal ID* + *Year*	4	0.0	1.00
*Animal ID*	3	15.3	0.00
Male Model Set 2 (all include *Animal ID* + *Year*)	*K*	Δ AICc	*W*
Null	4	0.0	0.66
*Castrated*	5	1.6	0.29
*Season*	7	6.1	0.03
*Castrated + Season*	8	7.8	0.01
Male Model Set 3 (all include *Animal ID* + *Year*)	*K*	Δ AICc	*W*
*Leu_D_ + Glx_D_ + Pro_D_*	7	0.0	0.17
*Leu_D_*	5	1.5	0.08
*Null*	4	5.8	0.01
*Bulk*	5	7.4	0.00
*Bulk_D_*	5	8.2	0.00

*K* is the number of parameters in the model, Δ AIC is the difference of AIC values from the best approximating model, and W is the AICc model weight. The subscript ‘D’ indicates that the δ^15^N value has been corrected for dietary (D) effects by subtraction of the Phe δ^15^N value. See Supplementary Table S8 for tables of full model selection results.

**Table 6:** Model parameter estimates for prediction of male muskoxen Diff_EM_ (the difference from expected mass, kg)

Fixed effects	Estimate	95% CI	*t*-value
Intercept	−104.34	−143.22, −65.49	−5.11
*Glx_D_*	−16.60	−30.06, −3.26	−2.37
*Leu_D_*	−10.55	−17.17, −3.87	−3.09
*Pro_D_*	14.16	2.70, 25.76	2.38
Random effects	SD	ICC	
*Animal ID*	11.5	0.101	
*Year*	22.7	0.397	
Residual	25.5		

Marginal pseudo-*R*^2^ = 0.238; conditional pseudo-*R*^2^ = 0.617.

The subscript ‘D’ indicates that the δ^15^N value has been corrected for dietary (D) effects by subtraction of the Phe δ^15^N value. ICC is intraclass correlation.

As predicted, Phe-corrected AA δ^15^N values were more sensitive to Diff_EM_ than bulk δ^15^N, which was not associated with Diff_EM_. The bulk δ^15^N value is a weighted average of all AAs and other N-containing compounds in the measured tissue or biological component, and as our results indicate, the δ^15^N values of specific AAs can be positively, negatively or not correlated with underweight status. This mixing of multiple δ^15^N signals in bulk dilutes the body condition signal present in individual AAs and, together with environmental and dietary noise, may explain why some studies fail to detect a signal of underweight status in the bulk δ^15^N value (Ben-David *et al.,* 1999; Kempster *et al.,* 2007; Gómez-Campos *et al.,* 2011).

**Figure 4:**
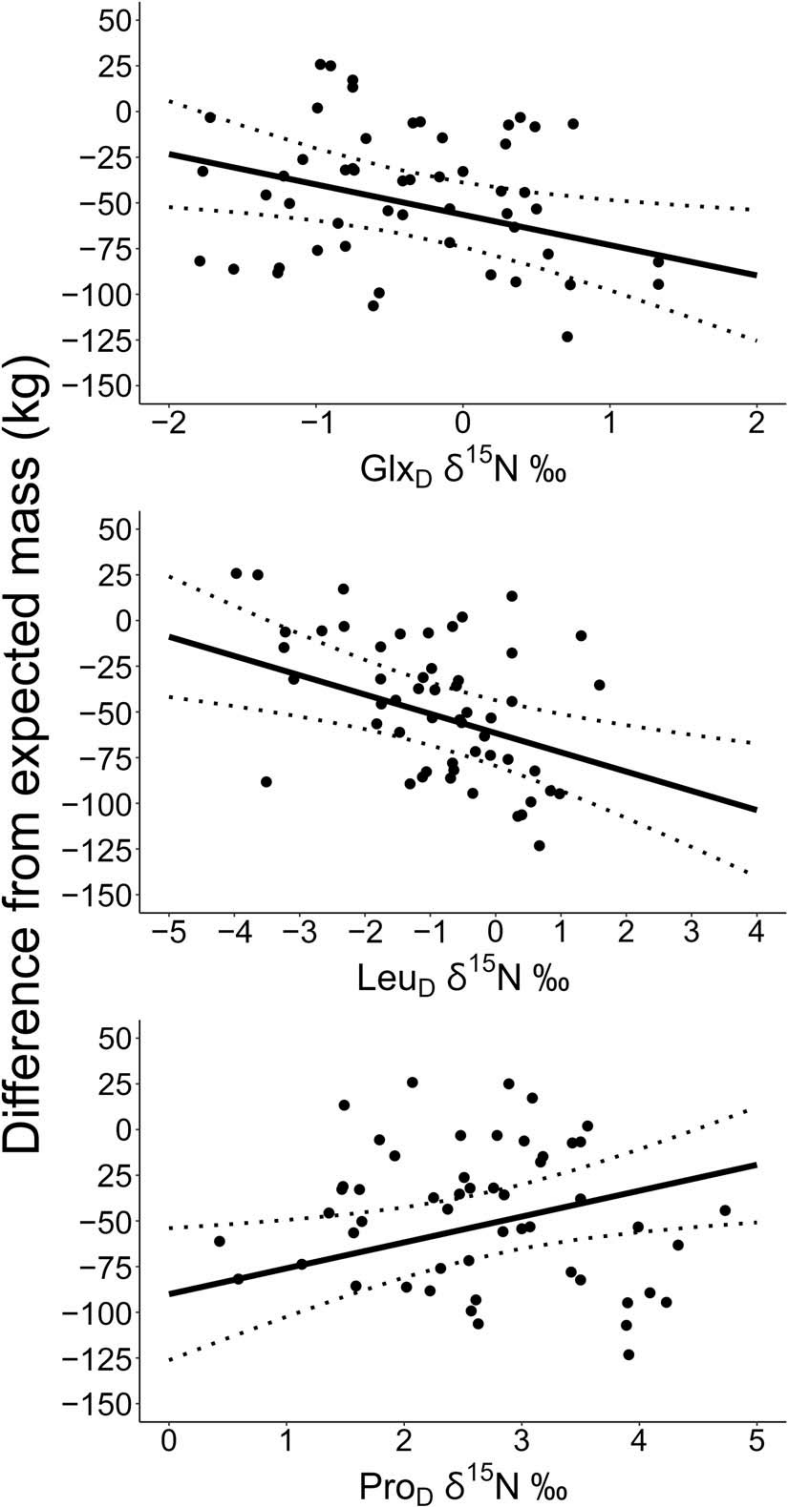
Predicted Diff_EM_ (the difference from expected mass, kg) for male muskoxen given their AA_D_ δ^15^N value. Dotted lines represent 95% CIs from model predictions. For each specific AA_D_ δ^15^N value, the other AA_D_ δ^15^N values were held at their mean. Points represent individual data points.

Because Glu is a central hub for transfer of amino groups in nitrogen metabolism (Newsholme *et al.,* 2003) and because the δ^15^N value of Glx_D_ is highly sensitive to trophic position, we predicted that it would be one of the most informative AAs in predicting Diff_EM_. Our prediction was based on numerous studies showing increased δ^15^N values of Glx_D_ with trophic level (Petzke *et al.,* 2005; McMahon and McCarthy, 2016; O’Connell, 2017; Ishikawa, 2018), demonstrating that ^15^N is retained in the Glx pool as nitrogen is consumed and excreted. Early studies established that transamination reactions, such as that catalysed by glutamine–oxaloacetic transaminase, prefer ^14^N (Macko *et al.,* 1986), leaving residual (non-waste) glutamate with higher ^15^N. We would expect ^15^N to further increase when the exit of nitrogen from the pool is unbalanced by dietary intake, such as in animals that are losing body mass. Our data upheld this prediction: Glx_D_ had a strong negative correlation with the Diff_EM_, consistent in both female and male muskoxen. Every 1‰ increase in Glx_D_ δ^15^N values represented 18.6 and 16.6 kg decline from Diff_EM_ for females and males, respectively. Like Glx_D_, Leu_D_ had a negative correlation with Diff_EM_, with every 1‰ increase in Leu_D_ δ^15^N values representing 7.9 and 10.6 kg declines from Diff_EM_ in females and males, respectively. This effect may result from the reamination of Leu through the reaction of Glu and α-ketoisocaproate (a keto acid) (Matthews *et al.,* 1981; Nissen and Haymond, 1981; Krishnamurti and Janssens, 1988; Tessari, 2017). As the δ^15^N value of Glx_D_ increased with a decrease in Diff_EM_, so did that of Leu_D_.

The next most predictive AAs, Asx_D_ and Ser_D_ in females and Pro_D_ in males, have highly interchangeable amino groups, but the direction of their association with Diff_EM_ differed from Glx_D_ and Leu_D_. In females, Asx_D_ and Ser_D_ were positively correlated with Diff_EM_*,* with a 1‰ increase representing 9.8 and 11.1 kg increases, respectively; and in males, Pro_D_ was positively correlated with Diff_EM_ with a 1‰ increase representing a 14.2-kg increase. These patterns are not consistent with the limited literature on AA δ^15^N changes with body condition; thus, additional studies are needed to confirm. The inverse associations with Asx_D_, Ser_D_ and Pro_D_ indicate that those serum pools become lighter during mass loss, which would indicate a preference for ^14^N at entry to the serum pool that is not counteracted by downstream effects. This could potentially occur with a shift to AA synthesis from endogenous keto-acids rather than supply from the diet, but this is speculative. Given the paucity of comparative data, more research is needed to verify that these sex-specific trends are consistent across studies and point to mechanisms that might be driving those differences.

Our study adds to a limited body of literature on using naturally abundant δ^15^N values of AAs to assess body condition in animals. In a study with pregnant bonnethead sharks (*Sphyrna tiburo*), δ^15^N values of Glx were lower in late term versus early term individuals (Shipley *et al.,* 2022), suggesting that late term sharks likely retained a greater proportion of ^14^N because they were in positive protein balance. Although this study on sharks focused on detecting gestational stage (i.e. growth), we also found that lower δ^15^N values of Glx correlated with individuals that were more massive. However, in a comparison of fasting and non-fasting free-range adult female southern elephant seals (*Mirounga leonina*), only glycine was found to have had a significantly higher δ^15^N value when elephant seals were in a fasting (i.e. underweight) state, a result that was inconsistent with our findings (Lübcker *et al.,* 2020). In a study where obese male Wistar rats (*Rattus norvegicus domestica*) were fed a calorie restricted diet, the rats had lower δ^15^N values of isoleucine, Leu and Val detected in their plasma than rats that had an ad libitum diet (Huneau *et al.,* 2019). The direction of change of δ^15^N values of Leu was similar to our study, but other findings were inconsistent. Finally, in a study with captive growing killifish (mummichogs, *Fundulus heteroclitus*), individuals in a negative AA imbalance state had higher δ^15^N values of alanine, Asx, Glx, isoleucine and Leu (with Phe used to correct for dietary δ^15^N) than individuals in a positive AA imbalance state (McMahon *et al.,* 2015), a finding that is partially consistent with ours (Glx, Leu) and partially inconsistent (Asx). Interestingly, neither of these studies nor our study found correlations between protein balance and the δ^15^N values of Thr, as has been hypothesized in other work (Wallace and Hedges, 2016). Direct comparison between different studies should be taken with some caution considering that different causes for changes in body condition (e.g. starvation, malnutrition, cachexia, growth, pregnancy) can affect how body stores of macronutrients are depleted or stored (Elango and Ball, 2016; Wu, 2016; Olson *et al.,* 2020).

A goal of this research was to better understand how δ^15^N values of AAs can be used to assess body condition in populations of animals, especially where traditional methods of examination are infeasible or impossible. Conditions where this method would be useful are when researchers only have a limited tissue sample, such as from hunter harvest, museum specimens or animal remains; when researchers have limited time with an animal, such as when it is anaesthetized in the field; or when an animal is too large to bring to a facility and it is infeasible to bring large measuring devices to it. Additionally, this method could be useful in archaeological studies, when bones or other preserved tissue are uncovered or exist in archived collections, to better understand the body condition of past humans and/or animals. We suggest this method could be useful as a general assessment of body condition, e.g. normal versus very poor. Generally, we found muskoxen were predicted to be in severe underweight status when the δ^15^N values of Glx_D_ were above 0‰ (when accounting for the δ^15^N values of Asx_D_, Leu_D_ and Ser_D_ for females and Leu_D_ and Pro_D_ for males).

This study had unique strengths for identifying stable isotope measures of body condition. The dietary mineral deficiency encountered with this captive herd of muskoxen was successfully resolved. However, this health problem in the muskoxen herd provided a valuable opportunity to evaluate how δ^15^N values of AAs were affected by underweight status in a large-bodied ruminant. Furthermore, we had access to data from a healthy muskoxen population from the same stock over a different temporal period, providing reference body mass data. Our study also had some limitations. Sample collection during a disease investigation often focuses on the clinical needs of individuals. For some muskoxen, there were fewer serum samples available and/or variable temporal gaps between samples. This made it challenging to monitor the body condition changes of individuals over time. Furthermore, we did not have a clear ‘healthy’ baseline for those individuals prior to 2010, as records suggest the subclinical effects of the dietary trace mineral deficiencies developed over a relatively long period of time prior to the 2010 morbidity–mortality event. To overcome this limitation, we used subsequently collected growth data from healthy muskoxen of the same stock to model body mass based on sex and age, and thus characterize the mass status of affected individuals relative to those norms. Further, there were potential differences in diet due to muskox dominance status and access to preferred foods, which we accounted for by adjusting all data relative to the δ^15^N value of Phe.

In conclusion, we found that δ^15^N values of AAs were associated with Diff_EM_ (the difference from expected mass, kg) in captive muskoxen, suggesting that they could be useful biomarkers of body condition in free-living herbivores. Our models using δ^15^N values of AAs were able to predict Diff_EM_ in muskoxen with relatively high accuracy. The results from this study could be a tool for wildlife researchers, veterinarians, physicians and archaeologists to evaluate protein condition in wildlife and domestic animals, and possibly humans. Future research is needed to better understand changes in AA δ^15^N values with protein status, including growth and body condition changes, in a more complete and diverse range of taxa.

## Supplementary Material

Web_Material_coag080

## Data Availability

Data that support the findings in this study are available on Dryad at https://doi.org/10.5061/dryad.rv15dv4pp.
