# Supplementary material for: Amino acid δ15N values are associated with underweight status in a captive population of muskoxen
Source: Conserv Physiol. 2026 Oct 22;14(1):coag080. doi: 10.1093/conphys/coag080 (PMC13688045; doi:10.1093/conphys/coag080)
Supplement: Supplementary file 1 [file web_material_coag080.zip › Savory_et_al_SupplementaryMaterials_18Aug26.docx]

Supplemental Information 1

Table SI.1. Known δ^15^N values of AAs used in mixed AA standards to correct and check sample δ^15^N values.

| **Standard** | **Component** | **δ^15^N** |
| --- | --- | --- |
| AA_1_ | Asp | -1.4 |
| AA_1_ | Glu | -3.5 |
| AA_1_ | Leu | +8.7 |
| AA_1_ | Phe | +9.6 |
| AA_1_ | Pro | -0.3 |
| AA_1_ | Ser | +2.3 |
| AA_1_ | Thr | -2.8 |
| AA_1_ | Val | +22.0 |
| AA_2_ | Asp | -5.3 |
| AA_2_ | Glu | -4.4 |
| AA_2_ | Leu | +2.5 |
| AA_2_ | Phe | +3.9 |
| AA_2_ | Pro | -6.7 |
| AA_2_ | Ser | -1.4 |
| AA_2_ | Thr | -1.7 |
| AA_2_ | Val | -0.7 |
| — | caffeine | -2.5 |
| — | norleucine #1 | +8.6 |
| — | norleucine #2 | +19.3 |

Supplemental Information 2

We modeled how different variables affected the δ^15^N values of Phe from serum of female and male muskoxen using general linear mixed-effect regression using the R package *lme4*. Our full additive models for female and male muskoxen included *Animal ID* as a random effect and *Diff_EM_*, *Age Class*, *Year* (each year treated as a categorical variable), *Season* (each season treated as a categorical variable), and *Pregnant* (only for the female model) as fixed effects (see **Methods—Body Condition Models** for variable descriptions). We considered a variable to have a significant effect if the 95% confidence interval did not overlap zero. For both female and male models, *Diff_EM_* had no significant effect on δ^15^N values of Phe (Table SI.1.1, SI.1.2). For the female model, we found that multiple years, except 2008 and 2010, and all seasons were significantly different from the intercept (Table SI.1.1). For the male model, we found that the years 2015 and 2016, and two seasons, late winter and spring and summer, were significantly different from the intercept (Table SI.1.2). The random effect of *Animal ID* accounted for 61% and 44% of the model error for the female and male models, respectively (Table SI.2.1, SI.2.2).

We used two-sided F-test for paired samples to test for significant differences in the variances between AA δ^15^N values uncorrected and corrected with Phe δ^15^N values. We found no significant differences (α level = 0.05) between the two sets of data for all AAs from female and male muskoxen (Tables SI.2.3, SI.2.4).

Table SI.2.1 Model parameter estimates for prediction of female muskoxen δ^15^N values of Phe from serum. ICC is intraclass correlation.

| Fixed Effects | Estimate | 95% CI | t-value |
| --- | --- | --- | --- |
| Intercept | 9.33 | 8.62, 10.05 | 25.57 |
| *Diff_EM_* | 0.00 | -0.01, 0.00 | -0.41 |
| *Age Class: subadult* | 0.02 | -0.41, 0.44 | 0.07 |
| *Year: 2008* | 0.46 | -0.20, 1.12 | 1.37 |
| *Year: 2009* | 0.92 | 0.07, 1.78 | 2.11 |
| *Year: 2010* | 0.55 | -0.12, 1.22 | 1.60 |
| *Year: 2011* | 1.24 | 0.50, 1.98 | 3.30 |
| *Year: 2012* | 0.80 | 0.12, 1.49 | 2.30 |
| *Year: 2013* | 0.74 | 0.03, 1.45 | 2.06 |
| *Year: 2014* | 1.45 | 0.72, 2.18 | 3.87 |
| *Year: 2015* | 1.40 | 0.70, 2.10 | 3.93 |
| *Year: 2016* | 1.09 | 0.35, 1.82 | 2.89 |
| *Season: early winter* | -1.09 | -1.47, -0.71 | -5.57 |
| *Season: late winter* | -1.85 | -2.40, -1.30 | -6.56 |
| *Season: spring and summer* | -1.11 | -1.43, -0.79 | -6.79 |
| *Pregnant: yes* | -0.02 | -0.43, 0.39 | -0.10 |
|  |  |  |  |
| Radom Effects | SD | ICC |  |
| *Animal ID* | 0.65 | 0.61 |  |
| Residual | 0.51 |  |  |

Table SI.2.2 Model parameter estimates for prediction of male muskoxen δ^15^N values of Phe from serum. ICC is intraclass correlation.

| Fixed Effects | Estimate | 95% CI | t-value |
| --- | --- | --- | --- |
| Intercept | 8.69 | 7.66, 9.72 | 16.49 |
| *Diff_EM_* | 0.00 | -0.01, 0.00 | -1.00 |
| *Age Class (subadult)* | 0.57 | -0.03, 1.18 | 1.85 |
| *Year: 2008* | 0.73 | -0.23, 1.70 | 1.49 |
| *Year: 2009* | 0.30 | -0.66, 1.26 | 0.62 |
| *Year: 2010* | 0.10 | -0.94, 1.13 | 0.18 |
| *Year: 2011* | 0.38 | -0.70, 1.46 | 0.69 |
| *Year: 2012* | 0.15 | -1.22, 1.51 | 0.21 |
| *Year: 2013* | 0.63 | -0.61, 1.86 | 1.00 |
| *Year: 2014* | 0.87 | -0.23, 1.97 | 1.55 |
| *Year: 2015* | 1.08 | 0.06, 2.09 | 2.08 |
| *Year: 2016* | 1.26 | 0.22, 2.31 | 2.37 |
| *Season (early winter)* | -0.50 | -1.10, 0.11 | -1.60 |
| *Season (late winter)* | -1.13 | -1.81, -0.46 | -3.30 |
| *Season (spring and summer)* | -0.77 | -1.43, -0.12 | -2.32 |
|  |  |  |  |
| Radom Effects | SD | ICC |  |
| *Animal ID* | 0.56 | 0.44 |  |
| Residual | 0.63 |  |  |

Table SI.2.3 Results of two-sided F tests comparing the variances between the δ^15^N values of amino acids (AAs) from female muskoxen serum with no Phe correction and with Phe correction.

|  | Variance | |  |  |
| --- | --- | --- | --- | --- |
| AA | No Phe Correction | Phe Correction | *F*_(1, 102)_ | *p* |
| Asx | 1.5 | 1.5 | 1.0 | 0.84 |
| Glx | 0.8 | 0.6 | 1.4 | 0.11 |
| Leu | 2.8 | 2.6 | 1.1 | 0.63 |
| Pro | 2.0 | 1.7 | 1.2 | 0.30 |
| Ser | 1.4 | 1.1 | 1.3 | 0.20 |
| Thr | 1.8 | 2.2 | 0.8 | 0.32 |
| Val | 1.2 | 1.0 | 1.3 | 0.25 |

Table SI.2.4 Results of two-sided F tests comparing the variances between the δ^15^N values of amino acids (AAs) from male muskoxen serum with no Phe correction and with Phe correction.

|  | Variance | |  |  |
| --- | --- | --- | --- | --- |
| AA | No Phe Correction | Phe Correction | *F*_(1, 55)_ | *p* |
| Asx | 0.9 | 1.6 | 0.6 | 0.06 |
| Glx | 0.8 | 0.9 | 0.9 | 0.55 |
| Leu | 2.1 | 2.0 | 1.0 | 0.93 |
| Pro | 0.9 | 1.3 | 0.7 | 0.18 |
| Ser | 2.4 | 1.8 | 1.3 | 0.31 |
| Thr | 1.9 | 2.2 | 0.8 | 0.55 |
| Val | 1.0 | 0.9 | 1.0 | 0.96 |

Supplemental Information 3

We evaluated how each AA δ^15^N value was affected by study variables for females and males with general linear mixed-effect regression using R package lme4 (Bates et al. 2015). We assessed two sets of models in a hierarchical approach with AICc to build our final models for female and male muskoxen for each δ^15^N value. The equation for the full model was: δ^15^N value = β_0_ + βX + θX + ε; where β_0_ is the intercept, βX is a vector of fixed effects, θX is a vector random effects, and ε is the residual error. In Model Set 1, we evaluated two additive models for each sex with random effects, Animal ID and Animal ID + Year. Animal ID represented unique individuals and Year was a categorical variable for each year of the study (2007–2016). All random effect models included Animal ID as a variable to prevent pseudo-replication. For model selection in Model Set 1, we used restricted maximum likelihood to fit models and assess model selection. In Model Set 2, we evaluated 13 mixed effect models for each sex including additive and interactive fixed effects with variables that potentially affected muskoxen δ^15^N values including the random effect variables selected from the best approximating model from Model Set 1. Models of both sexes included Diff_EM_ (the difference from expected mass, kg) and Age Class as variables. We included Pregnant as a variable in female models and included Castrated in male models. We also included a model with only random effects to assess as a null model. For model selection in Model Set 2, we used maximum likelihood to fit models and assess model selection. We checked models for meeting normality and homoscedasticity assumptions by visual examination of quantile-quantile plots and plotting model fits against model residuals. Results of model selection using AICc of linear mixed-effect models of female and male muskoxen with δ^15^N values as the response. Diff_EM_ is the difference from expected mass, kg. K is number of parameters, Δ AIC is the difference of AIC values from the best approximating model, and W is AICc model weight. The subscript “D” indicates that the δ^15^N value has been corrected for dietary (D) effects by subtraction of the Phe δ^15^N value.

Table SI.3a. Model selection of female muskoxen models with bulk δ^15^N values as the response variable.

| Random Effects Models | K | Δ AICc | W |
| --- | --- | --- | --- |
| *Animal ID* | 3 | 0.0 | 0.62 |
| *Animal ID* + *Year* | 4 | 1.0 | 0.38 |
|  |  |  |  |
| Fixed Effects Models (all include *Animal ID* as random effects) | K | Δ AICc | W |
| Null | 3 | 0.0 | 0.35 |
| *Age Class* | 4 | 1.4 | 0.17 |
| *Diff_EM_* | 4 | 2.1 | 0.12 |
| *Pregnant* | 4 | 2.2 | 0.12 |
| Diff_EM_ + *Age Class* | 5 | 3.4 | 0.06 |
| *Pregnant* + *Age Class* | 5 | 3.6 | 0.06 |
| *Diff_EM_* + *Pregnant* | 5 | 4.3 | 0.04 |
| *Diff_EM_* + *Age Class* + *Diff_EM_* : *Age Class* | 6 | 5.4 | 0.02 |
| *Diff_EM_* + *Age Class* + *Pregnant* | 6 | 5.7 | 0.02 |
| *Diff_EM_* + *Pregnant* + *Diff_EM_* : *Pregnant* | 6 | 6.3 | 0.01 |
| *Diff_EM_* + *Age Class* + *Pregnant* + *Diff_EM_* : *Age Class* | 7 | 7.7 | 0.01 |
| *Diff_EM_* + *Age Class* + *Pregnant* + *Diff_EM_* : *Pregnant* | 7 | 7.8 | 0.01 |
| *Diff_EM_* + *Age Class* + *Pregnant* + *Diff_EM_* : *Age Class* + *Diff_EM_* : *Pregnant* | 8 | 9.5 | 0.00 |

Table SI.3b. Model selection of female muskoxen models with bulk_D_ δ^15^N values as the response variable.

| Random Effects Models | K | Δ AICc | W |
| --- | --- | --- | --- |
| *Animal ID* | 3 | 0.0 | 0.76 |
| *Animal ID* + *Year* | 4 | 2.3 | 0.24 |
|  |  |  |  |
| Fixed Effects Models (all include *Animal ID* as random effects) | K | Δ AICc | W |
| *Pregnant* | 4 | 0.0 | 0.20 |
| Null | 3 | 0.4 | 0.16 |
| *Diff_EM_* + *Age Class* + *Pregnant* + *Diff_EM_* : *Age Class* | 7 | 0.6 | 0.15 |
| *Diff_EM_* + *Age Class* + *Pregnant* + *Diff_EM_* : *Age Class* + *Diff_EM_* : *Pregnant* | 8 | 1.8 | 0.08 |
| *Pregnant* + *Age Class* | 5 | 1.8 | 0.08 |
| *Diff_EM_* + *Pregnant* | 5 | 2.2 | 0.07 |
| *Age Class* | 4 | 2.3 | 0.06 |
| *Diff_EM_* | 4 | 2.4 | 0.06 |
| *Diff_EM_* + *Age Class* + *Diff_EM_* : *Age Class* | 6 | 2.5 | 0.06 |
| *Diff_EM_* + *Age Class* + *Pregnant* | 6 | 4.0 | 0.03 |
| *Diff_EM_* + *Age Class* | 5 | 4.2 | 0.02 |
| *Diff_EM_* + *Pregnant* + *Diff_EM_* : *Pregnant* | 6 | 4.4 | 0.02 |
| *Diff_EM_* + *Age Class* + *Pregnant* + *Diff_EM_* : *Pregnant* | 7 | 6.3 | 0.01 |

Table SI.3c. Model selection of female muskoxen models with Asx_D_ δ^15^N values as the response variable.

| Random Effects Models | K | Δ AICc | W |
| --- | --- | --- | --- |
| *Animal ID* | 3 | 0.0 | 0.75 |
| *Animal ID* + *Year* | 4 | 2.2 | 0.25 |
|  |  |  |  |
| Fixed Effects Models (all include *Animal ID* as random effects) | K | Δ AICc | W |
| *Age Class* | 4 | 0.0 | 0.22 |
| Null | 3 | 0.1 | 0.22 |
| *Diff_EM_* + *Age Class* | 5 | 1.5 | 0.11 |
| *Diff_EM_* | 4 | 1.5 | 0.10 |
| *Pregnant* | 4 | 2.0 | 0.08 |
| *Pregnant* + *Age Class* | 5 | 2.1 | 0.08 |
| *Diff_EM_* + *Age Class* + *Diff_EM_* : *Age Class* | 6 | 2.9 | 0.05 |
| *Diff_EM_* + *Pregnant* | 5 | 3.7 | 0.04 |
| *Diff_EM_* + *Age Class* + *Pregnant* | 6 | 3.7 | 0.04 |
| *Diff_EM_* + *Pregnant* + *Diff_EM_* : *Pregnant* | 6 | 4.5 | 0.02 |
| *Diff_EM_* + *Age Class* + Pregnant + *Diff_EM_* : *Pregnant* | 7 | 5.1 | 0.02 |
| *Diff_EM_* + *Age Class* + *Pregnant* + *Diff_EM_* : *Age Class* | 7 | 5.1 | 0.02 |
| *Diff_EM_* + *Age Class* + *Pregnant* + *Diff_EM_* : *Age Class* + *Diff_EM_* : *Pregnant* | 8 | 7.0 | 0.01 |

Table SI.3d. Model selection of female muskoxen models with Glx_D_ δ^15^N values as the response variable.

| Random Effects Models | K | Δ AICc | W |
| --- | --- | --- | --- |
| *Animal ID* | 3 | 0.0 | 0.54 |
| *Animal ID* + *Year* | 4 | 0.3 | 0.46 |
|  |  |  |  |
| Fixed Effects Models (all include *Animal ID* as random effects) | K | Δ AICc | W |
| Null | 3 | 0.0 | 0.21 |
| *Age Class* | 4 | 0.1 | 0.20 |
| *Diff_EM_* | 4 | 1.2 | 0.12 |
| *Diff_EM_* + *Age Class* | 5 | 1.5 | 0.10 |
| *Pregnant* | 4 | 2.1 | 0.07 |
| *Pregnant* + *Age Class* | 5 | 2.2 | 0.07 |
| *Diff_EM_* + *Age Class* + *Diff_EM_* : *Age Class* | 6 | 2.4 | 0.06 |
| *Diff_EM_* + *Pregnant* | 5 | 3.0 | 0.05 |
| *Diff_EM_* + *Age Class* + *Pregnant* | 6 | 3.2 | 0.04 |
| *Diff_EM_* + *Age Class* + *Pregnant* + *Diff_EM_* : *Age Class* | 7 | 3.4 | 0.04 |
| *Diff_EM_* + *Pregnant* + *Diff_EM_* : *Pregnant* | 6 | 5.0 | 0.02 |
| *Diff_EM_* + *Age Class* + *Pregnant* + *Diff_EM_* : *Pregnant* | 7 | 5.4 | 0.01 |
| *Diff_EM_* + *Age Class* + *Pregnant* + *Diff_EM_* : *Age Class* + *Diff_EM_* : *Pregnant* | 8 | 5.7 | 0.01 |

Table SI.3e. Model selection of female muskoxen models with Leu_D_ δ^15^N values as the response variable.

| Random Effects Models | K | Δ AICc | W |
| --- | --- | --- | --- |
| *Animal ID* | 3 | 0.0 | 0.62 |
| *Animal ID* + *Year* | 4 | 1.0 | 0.38 |
|  |  |  |  |
| Fixed Effects Models (all include *Animal ID* as random effects) | K | Δ AICc | W |
| *Diff_EM_* + *Pregnant* + *Diff_EM_* : *Pregnant* | 6 | 0.0 | 0.30 |
| *Diff_EM_* + *Age Class* + *Pregnant* + *Diff_EM_* : *Age Class* | 7 | 0.7 | 0.21 |
| *Diff_EM_* + *Pregnant* | 5 | 1.5 | 0.14 |
| *Diff_EM_* + *Age Class* + *Pregnant* + *Diff_EM_* : *Age Class* + *Diff_EM_* : *Pregnant* | 8 | 1.6 | 0.13 |
| *Diff_EM_* + *Age Class* + *Pregnant* + *Diff_EM_* : *Pregnant* | 7 | 1.7 | 0.13 |
| *Diff_EM_* + *Age Class* + *Pregnant* | 6 | 2.5 | 0.08 |
| *Diff_EM_* | 4 | 7.6 | 0.01 |
| *Diff_EM_* + *Age Class* | 5 | 9.4 | 0.00 |
| *Diff_EM_* + *Age Class* + *Diff_EM_* : *Age Class* | 6 | 10.9 | 0.00 |
| *Pregnant* | 4 | 11.7 | 0.00 |
| Null | 3 | 11.9 | 0.00 |
| *Pregnant* + *Age Class* | 5 | 13.4 | 0.00 |
| *Age Class* | 4 | 13.8 | 0.00 |

Table SI.3f. Model selection of female muskoxen models with Pro_D_ δ^15^N values as the response variable.

| Random Effects Models | K | Δ AICc | W |
| --- | --- | --- | --- |
| *Animal ID* | 3 | 0.0 | 0.66 |
| *Animal ID* + *Year* | 4 | 1.3 | 0.34 |
|  |  |  |  |
| Fixed Effects Models (all include *Animal ID* as random effects) | K | Δ AICc | W |
| *Pregnant* | 4 | 0.0 | 0.41 |
| *Diff_EM_* + *Age Class* + *Pregnant* + *Diff_EM_* : *Age Class* | 7 | 2.1 | 0.15 |
| *Pregnant* + *Age Class* | 5 | 2.1 | 0.14 |
| *Diff_EM_* + *Pregnant* | 5 | 2.2 | 0.14 |
| *Diff_EM_* + *Pregnant* + *Diff_EM_* : *Pregnant* | 6 | 4.3 | 0.05 |
| *Diff_EM_* + *Age Class* + *Pregnant* | 6 | 4.3 | 0.05 |
| *Diff_EM_* + *Age Class* + *Pregnant* + *Diff_EM_* : *Age Class* + *Diff_EM_* : *Pregnant* | 8 | 4.3 | 0.05 |
| *Diff_EM_* + *Age Class* + *Pregnant* + *Diff_EM_* : *Pregnant* | 7 | 6.5 | 0.02 |
| Null | 3 | 11.7 | 0.00 |
| *Diff_EM_* | 4 | 12.2 | 0.00 |
| *Age Class* | 4 | 13.8 | 0.00 |
| *Diff_EM_* + *Age Class* | 5 | 14.4 | 0.00 |
| *Diff_EM_* + *Age Class* + *Diff_EM_* : *Age Class* | 6 | 15.7 | 0.00 |

Table SI.3h. Model selection of female muskoxen models with Ser_D_ δ^15^N values as the response variable.

| Random Effects Models | K | Δ AICc | W |
| --- | --- | --- | --- |
| *Animal ID* | 3 | 0.0 | 0.72 |
| *Animal ID* + *Year* | 4 | 1.9 | 0.28 |
|  |  |  |  |
| Fixed Effects Models (all include *Animal ID* as random effects) | K | Δ AICc | W |
| *Diff_EM_* + *Age Class* + *Diff_EM_* : *Age Class* | 6 | 0.0 | 0.35 |
| *Diff_EM_* | 4 | 1.3 | 0.18 |
| *Diff_EM_* + *Age Class* + *Pregnant* + *Diff_EM_* : *Age Class* + *Diff_EM_* : *Pregnant* | 8 | 2.0 | 0.13 |
| *Diff_EM_* + *Age Class* + *Pregnant* + *Diff_EM_* : *Age Class* | 7 | 2.2 | 0.12 |
| *Diff_EM_* + *Pregnant* | 5 | 3.1 | 0.08 |
| *Diff_EM_* + *Age Class* | 5 | 3.4 | 0.06 |
| *Diff_EM_* + *Pregnant* + *Diff_EM_* : *Pregnant* | 6 | 4.8 | 0.03 |
| *Diff_EM_* + *Age Class* + *Pregnant* | 6 | 5.2 | 0.03 |
| *Diff_EM_* + *Age Class* + *Pregnant* + *Diff_EM_* : *Pregnant* | 7 | 7.0 | 0.01 |
| Null | 3 | 7.5 | 0.01 |
| *Pregnant* | 4 | 9.5 | 0.00 |
| *Age Class* | 4 | 9.6 | 0.00 |
| *Pregnant* + *Age Class* | 5 | 11.6 | 0.00 |

Table SI.3i. Model selection of female muskoxen models with Thr_D_ δ^15^N values as the response variable.

| Random Effects Models | K | Δ AICc | W |
| --- | --- | --- | --- |
| *Animal ID* + *Year* | 4 | 0.0 | 0.88 |
| *Animal ID* | 3 | 4.0 | 0.12 |
|  |  |  |  |
| Fixed Effects Models (all include *Animal ID* + *Year* as random effects) | K | Δ AICc | W |
| Null | 4 | 0.0 | 0.37 |
| *Age Class* | 5 | 2.0 | 0.14 |
| *Pregnant* | 5 | 2.1 | 0.13 |
| *Diff_EM_* | 5 | 2.2 | 0.12 |
| *Pregnant* + *Age Class* | 6 | 4.1 | 0.05 |
| *Diff_EM_* + *Age Class* | 6 | 4.2 | 0.04 |
| *Diff_EM_* + *Pregnant* | 6 | 4.3 | 0.04 |
| *Diff_EM_* + *Age Class* + *Diff_EM_* : *Age Class* | 7 | 4.5 | 0.04 |
| *Diff_EM_* + *Age Class* + Pregnant + *Diff_EM_* : *Age Class* | 8 | 6.1 | 0.02 |
| *Diff_EM_* + *Age Class* + *Pregnant* | 7 | 6.3 | 0.02 |
| *Diff_EM_* + *Pregnant* + *Diff_EM_* : *Pregnant* | 7 | 6.5 | 0.01 |
| *Diff_EM_* + *Age Class* + *Pregnant* + *Diff_EM_* : *Age Class* + *Diff_EM_* : *Pregnant* | 9 | 8.4 | 0.01 |
| *Diff_EM_* + *Age Class* + *Pregnant* + *Diff_EM_* : *Pregnant* | 8 | 8.6 | 0.01 |

Table SI.3j. Model selection of female muskoxen models with Val_D_ δ^15^N values as the response variable.

| Random Effects Models | K | Δ AICc | W |
| --- | --- | --- | --- |
| *Animal ID* | 3 | 0.0 | 0.72 |
| *Animal ID* + *Year* | 4 | 1.9 | 0.28 |
|  |  |  |  |
| Fixed Effects Models (all include *Animal ID* as random effects) | K | Δ AICc | W |
| *Pregnant* | 4 | 0.0 | 0.28 |
| *Diff_EM_* + *Pregnant* | 5 | 1.0 | 0.17 |
| Null | 3 | 1.1 | 0.16 |
| *Pregnant* + *Age Class* | 5 | 2.2 | 0.09 |
| *Diff_EM_* | 4 | 3.2 | 0.06 |
| *Age Class* | 4 | 3.2 | 0.06 |
| *Diff_EM_* + *Pregnant* + *Diff_EM_* : *Pregnant* | 6 | 3.3 | 0.06 |
| *Diff_EM_* + *Age Class* + *Pregnant* | 6 | 3.3 | 0.05 |
| *Diff_EM_* + *Age Class* + *Pregnant* + *Diff_EM_* : *Age Class* | 7 | 5.0 | 0.02 |
| *Diff_EM_* + *Age Class* | 5 | 5.4 | 0.02 |
| *Diff_EM_* + *Age Class* + *Pregnant* + *Diff_EM_* : *Pregnant* | 7 | 5.6 | 0.02 |
| *Diff_EM_* + *Age Class* + *Pregnant* + *Diff_EM_* : *Age Class* + *Diff_EM_* : *Pregnant* | 8 | 7.4 | 0.01 |
| *Diff_EM_* + *Age Class* + *Diff_EM_* : *Age Class* | 6 | 7.6 | 0.01 |

Table SI.3k. Model selection of male muskoxen models with bulk δ^15^N values as the response variable.

| Random Effects Models | K | Δ AICc | W |
| --- | --- | --- | --- |
| *Animal ID* | 3 | 0.0 | 0.62 |
| *Animal ID* + *Year* | 4 | 1.0 | 0.38 |
|  |  |  |  |
| Fixed Effects Models (all include *Animal ID* as random effects) | K | Δ AICc | W |
| Null | 3 | 0.0 | 0.31 |
| *Age Class* | 4 | 1.1 | 0.18 |
| *Diff_EM_* | 4 | 1.8 | 0.12 |
| *Castrated* | 4 | 2.2 | 0.10 |
| *Age Class* + *Castrated* | 5 | 3.0 | 0.07 |
| *Diff_EM_* + *Age Class* | 5 | 3.0 | 0.07 |
| *Diff_EM_* + *Castrated* | 5 | 4.2 | 0.04 |
| *Diff_EM_* + *Age Class* + *Castrated* | 6 | 4.8 | 0.03 |
| *Diff_EM_* + *Age Class* + *Diff_EM_* : *Age Class* | 6 | 4.8 | 0.03 |
| *Diff_EM_* + *Castrated* + *Diff_EM_* : *Castrated* | 6 | 5.1 | 0.02 |
| *Diff_EM_* + *Age Class* + *Castrated* + *Diff_EM_* : *Castrated* | 7 | 5.7 | 0.02 |
| *Diff_EM_* + *Age Class* + *Castrated* + *Diff_EM_* : *Age Class* | 7 | 6.6 | 0.01 |
| *Diff_EM_* + *Age Class* + *Castrated* + *Diff_EM_* : *Age Class* + *Diff_EM_* : *Castrated* | 8 | 8.2 | 0.00 |

Table SI.3l. Model selection of male muskoxen models with bulk_D_ δ^15^N values as the response variable.

| Random Effects Models | K | Δ AICc | W |
| --- | --- | --- | --- |
| *Animal ID* | 3 | 0.0 | 0.76 |
| *Animal ID* + *Year* | 4 | 2.3 | 0.24 |
|  |  |  |  |
| Fixed Effects Models (all include *Animal ID* as random effects) | K | Δ AICc | W |
| Null | 3 | 0.0 | 0.24 |
| *Diff_EM_* | 4 | 0.4 | 0.19 |
| *Age Class* | 4 | 1.4 | 0.12 |
| *Diff_EM_* + *Age Class* | 5 | 1.8 | 0.10 |
| *Castrated* | 4 | 2.0 | 0.09 |
| *Diff_EM_* + *Castrated* | 5 | 2.4 | 0.07 |
| *Diff_EM_* + *Castrated* + *Diff_EM_* : *Castrated* | 6 | 2.8 | 0.06 |
| *Age Class* + *Castrated* | 5 | 3.8 | 0.04 |
| *Diff_EM_* + *Age Class* + *Diff_EM_* : *Age Class* | 6 | 4.2 | 0.03 |
| *Diff_EM_* + *Age Class* + *Castrated* | 6 | 4.3 | 0.03 |
| *Diff_EM_* + *Age Class* + *Castrated* + *Age Class* + *Diff_EM_* : *Castrated* | 7 | 5.0 | 0.02 |
| *Diff_EM_* + *Age Class* + *Castrated* + *Diff_EM_* : *Age Class* + *Diff_EM_* : *Castrated* | 8 | 5.8 | 0.01 |
| *Diff_EM_* + *Age Class* + *Castrated* + *Diff_EM_* : *Age Class* | 7 | 6.8 | 0.01 |

Table SI.3m. Model selection of male muskoxen models with Asx_D_ δ^15^N values as the response variable.

| Random Effects Models | K | Δ AICc | W |
| --- | --- | --- | --- |
| *Animal ID* | 3 | 0.0 | 0.75 |
| *Animal ID* + *Year* | 4 | 2.2 | 0.25 |
|  |  |  |  |
| Fixed Effects Models (all include *Animal ID* as random effects) | K | Δ AICc | W |
| *Diff_EM_* | 4 | 0.0 | 0.41 |
| *Diff_EM_* + *Age Class* | 5 | 2.0 | 0.15 |
| *Diff_EM_* + *Castrated* | 5 | 2.4 | 0.12 |
| *Diff_EM_* + *Age Class* + *Diff_EM_* : *Age Class* | 6 | 3.6 | 0.07 |
| *Diff_EM_* + *Age Class* + *Castrated* + *Diff_EM_* : *Age Class* + *Diff_EM_* : *Castrated* | 8 | 3.7 | 0.07 |
| *Diff_EM_* + *Age Class* + *Castrated* | 6 | 4.4 | 0.04 |
| *Diff_EM_* + *Castrated* + *Diff_EM_* : *Castrated* | 6 | 4.8 | 0.04 |
| Null | 3 | 4.9 | 0.04 |
| *Diff_EM_* + *Age Class* + *Castrated* + *Diff_EM_* : *Age Class* | 7 | 5.9 | 0.02 |
| *Diff_EM_* + *Age Class* : *Castrated* + *Diff_EM_* : *Castrated* | 7 | 6.9 | 0.01 |
| *Age Class* | 4 | 7.0 | 0.01 |
| *Castrated* | 4 | 7.2 | 0.01 |
| *Age Class* + *Castrated* | 5 | 9.2 | 0.00 |

Table SI.3n. Model selection of male muskoxen models with Glx_D_ δ^15^N values as the response variable.

| Random Effects Models | K | Δ AICc | W |
| --- | --- | --- | --- |
| *Animal ID* | 3 | 0.0 | 0.54 |
| *Animal ID* + *Year* | 4 | 0.3 | 0.46 |
|  |  |  |  |
| Fixed Effects Models (all include *Animal ID* as random effects) | K | Δ AICc | W |
| *Diff_EM_* | 4 | 0.0 | 0.42 |
| *Diff_EM_* + *Age Class* | 5 | 2.3 | 0.13 |
| *Diff_EM_* + *Castrated* | 5 | 2.4 | 0.13 |
| Null | 3 | 3.4 | 0.08 |
| *Diff_EM_* + *Castrated* + *Diff_EM_* : *Castrated* | 6 | 4.3 | 0.05 |
| *Diff_EM_* + *Age Class* + *Castrated* | 6 | 4.5 | 0.04 |
| *Diff_EM_* + *Age Class* + *Diff_EM_* : *Age Class* | 6 | 4.7 | 0.04 |
| *Diff_EM_* + *Age Class* + *Castrated* + *Diff_EM_* : *Age Class* + *Diff_EM_* : *Castrated* | 8 | 5.0 | 0.03 |
| *Castrated* | 4 | 5.6 | 0.03 |
| *Age Class* | 4 | 5.7 | 0.02 |
| *Diff_EM_* + *Age Class* + *Castrated* + *Age Class* + *Diff_EM_* : *Castrated* | 7 | 6.6 | 0.02 |
| *Diff_EM_* + *Age Class* + *Castrated* + *Diff_EM_* : *Age Class* | 7 | 6.9 | 0.01 |
| *Age Class* + *Castrated* | 5 | 7.7 | 0.01 |

Table SI.3o. Model selection of male muskoxen models with Leu_D_ δ^15^N values as the response variable.

| Random Effects Models | K | Δ AICc | W |
| --- | --- | --- | --- |
| *Animal ID* | 3 | 0.0 | 0.72 |
| *Animal ID* + *Year* | 4 | 1.9 | 0.28 |
|  |  |  |  |
| Fixed Effects Models (all include *Animal ID* as random effects) | K | Δ AICc | W |
| *Diff_EM_* + *Age Class* | 5 | 0.0 | 0.29 |
| *Diff_EM_* + *Age Class* + *Castrated* | 6 | 0.5 | 0.23 |
| *Diff_EM_* | 4 | 1.9 | 0.11 |
| *Diff_EM_* + *Age Class* + *Diff_EM_* : *Age Class* | 6 | 2.1 | 0.10 |
| *Diff_EM_* + *Age Class* + *Castrated* + *Age Class* + *Diff_EM_* : *Castrated* | 7 | 2.8 | 0.07 |
| *Diff_EM_* + *Age Class* + *Castrated* + *Diff_EM_* : *Age Class* | 7 | 2.9 | 0.07 |
| *Diff_EM_* + *Castrated* | 5 | 4.2 | 0.04 |
| *Diff_EM_* + *Age Class* + *Castrated* + *Diff_EM_* : *Age Class* + *Diff_EM_* : *Castrated* | 8 | 5.5 | 0.02 |
| *Age Class* | 4 | 5.6 | 0.02 |
| *Diff_EM_* + *Castrated* + *Diff_EM_* : *Castrated* | 6 | 6.0 | 0.01 |
| *Age Class* + *Castrated* | 5 | 6.3 | 0.01 |
| Null | 3 | 6.7 | 0.01 |
| *Castrated* | 4 | 9.0 | 0.00 |

Table SI.3p. Model selection of male muskoxen models with Pro_D_ δ^15^N values as the response variable.

| Random Effects Models | K | Δ AICc | W |
| --- | --- | --- | --- |
| *Animal ID* | 3 | 0.0 | 0.71 |
| *Animal ID* + *Year* | 4 | 1.8 | 0.29 |
|  |  |  |  |
| Fixed Effects Models (all include *Animal ID* as random effects) | K | Δ AICc | W |
| *Diff_EM_* + *Castrated* + *Diff_EM_* : *Castrated* | 6 | 0.0 | 0.19 |
| Null | 3 | 0.6 | 0.14 |
| *Diff_EM_* + *Age Class* + *Castrated* + *Diff_EM_* : *Age Class* + *Diff_EM_* : *Castrated* | 8 | 0.6 | 0.14 |
| *Age Class* | 4 | 1.0 | 0.11 |
| *Diff_EM_* + *Age Class* + *Castrated* + *Age Class* + *Diff_EM_* : *Castrated* | 7 | 1.2 | 0.10 |
| *Diff_EM_* | 4 | 1.7 | 0.08 |
| *Diff_EM_* + *Age Class* | 5 | 2.1 | 0.06 |
| *Castrated* | 4 | 2.5 | 0.05 |
| *Age Class* + *Castrated* | 5 | 3.4 | 0.03 |
| *Diff_EM_* + *Age Class* + *Diff_EM_* : *Age Class* | 6 | 3.5 | 0.03 |
| *Diff_EM_* + *Castrated* | 5 | 3.6 | 0.03 |
| *Diff_EM_* + *Age Class* + *Castrated* | 6 | 4.6 | 0.02 |
| *Diff_EM_* + *Age Class* + *Castrated* + *Diff_EM_* : *Age Class* | 7 | 6.1 | 0.01 |

Table SI.3q. Model selection of male muskoxen models with Ser_D_ δ^15^N values as the response variable.

| Random Effects Models | K | Δ AICc | W |
| --- | --- | --- | --- |
| *Animal ID* + *Year* | 4 | 0.0 | 0.93 |
| *Animal ID* | 3 | 5.3 | 0.07 |
|  |  |  |  |
| Fixed Effects Models (all include *Animal ID* + *Year* as random effects) | K | Δ AICc | W |
| Null | 4 | 0.0 | 0.34 |
| *Age Class* | 5 | 1.0 | 0.21 |
| *Castrated* | 5 | 2.2 | 0.11 |
| *Diff_EM_* | 5 | 2.3 | 0.11 |
| *Age Class* + *Castrated* | 6 | 3.3 | 0.07 |
| *Diff_EM_* + *Age Class* | 6 | 3.5 | 0.06 |
| *Diff_EM_* + *Castrated* | 6 | 4.7 | 0.03 |
| *Diff_EM_* + *Age Class* + *Diff_EM_* : *Age Class* | 7 | 5.1 | 0.03 |
| *Diff_EM_* + *Age Class* + *Castrated* | 7 | 5.9 | 0.02 |
| *Diff_EM_* + *Castrated* + *Diff_EM_* : *Castrated* | 7 | 7.3 | 0.01 |
| *Diff_EM_* + *Age Class* + *Castrated* + *Diff_EM_* : *Age Class* | 8 | 7.7 | 0.01 |
| *Diff_EM_* + *Age Class* + *Castrated* + *Diff_EM_* : *Castrated* | 8 | 8.6 | 0.00 |
| *Diff_EM_* + *Age Class* + *Castrated* + *Diff_EM_* : *Age Class* + *Diff_EM_* : *Castrated* | 9 | 8.9 | 0.00 |

Table SI.3r. Model selection of male muskoxen models with Thr_D_ δ^15^N values as the response variable.

| Random Effects Models | K | Δ AICc | W |
| --- | --- | --- | --- |
| *Animal ID* + *Year* | 4 | 0.0 | 0.81 |
| *Animal ID* | 3 | 2.9 | 0.19 |
|  |  |  |  |
| Fixed Effects Models (all include *Animal ID* and *Year* as random effects) | K | Δ AICc | W |
| Null | 4 | 0.0 | 0.25 |
| *Age Class* | 5 | 0.8 | 0.16 |
| *Diff_EM_* + *Age Class* + *Diff_EM_* : *Age Class* | 7 | 1.6 | 0.11 |
| *Diff_EM_* | 5 | 2.0 | 0.09 |
| *Castrated* | 5 | 2.2 | 0.08 |
| *Diff_EM_* + *Castrated* + *Diff_EM_* : *Castrated* | 7 | 2.5 | 0.07 |
| *Age Class* + *Castrated* | 6 | 2.9 | 0.06 |
| *Diff_EM_* + *Age Class* + *Castrated* + *Diff_EM_* : *Castrated* | 8 | 3.2 | 0.05 |
| *Diff_EM_* + *Age Class* | 6 | 3.4 | 0.05 |
| *Diff_EM_* + *Age Class* + *Castrated* + *Diff_EM_* : *Age Class* | 8 | 4.3 | 0.03 |
| *Diff_EM_* + *Castrated* | 6 | 4.4 | 0.03 |
| *Diff_EM_* + *Age Class* + *Castrated* | 7 | 5.5 | 0.02 |
| *Diff_EM_* + *Age Class* + *Castrated* + *Diff_EM_* : *Age Class* + *Diff_EM_* : *Castrated* | 9 | 6.0 | 0.01 |

Table SI.3s. Model selection of male muskoxen models with Val_D_ δ^15^N values as the response variable.

| Random Effects Models | K | Δ AICc | W |
| --- | --- | --- | --- |
| *Animal ID* | 3 | 0.0 | 0.76 |
| *Animal ID* + *Year* | 4 | 2.3 | 0.24 |
|  |  |  |  |
| Fixed Effects Models (all include *Animal ID* as random effects) | K | Δ AICc | W |
| Null | 3 | 0.0 | 0.40 |
| *Diff_EM_* | 4 | 1.9 | 0.15 |
| *Castrated* | 4 | 2.2 | 0.13 |
| *Age Class* | 4 | 2.3 | 0.13 |
| *Diff_EM_* + *Castrated* | 5 | 4.2 | 0.05 |
| *Diff_EM_* + *Age Class* | 5 | 4.3 | 0.05 |
| *Age Class* + *Castrated* | 5 | 4.7 | 0.04 |
| *Diff_EM_* + *Age Class* + *Diff_EM_* : *Age Class* | 6 | 6.1 | 0.02 |
| *Diff_EM_* + *Castrated* + *Diff_EM_* : *Castrated* | 6 | 6.6 | 0.01 |
| *Diff_EM_* + *Age Class* + *Castrated* | 6 | 6.7 | 0.01 |
| *Diff_EM_* + *Age Class* + *Castrated* + *Diff_EM_* : *Age Class* | 7 | 8.7 | 0.01 |
| *Diff_EM_* + *Age Class* + *Castrated* + *Age Class* + *Diff_EM_* : *Castrated* | 7 | 9.2 | 0.00 |
| *Diff_EM_* + *Age Class* + *Castrated* + *Diff_EM_* : *Age Class* + *Diff_EM_* : *Castrated* | 8 | 11.0 | 0.00 |

Supplemental Information 4

Estimates from linear mixed-effect models of female and male muskoxen with δ^15^N values as the response. The subscript “D” indicates that the δ^15^N value has been corrected for dietary (D) effects by subtraction of the Phe δ^15^N value. β_0_ represents the model intercept. Random effects (Animal ID and Year) represented unique individuals and categorical variable for each year of the study (2007–2016).

Table SI.4a. Estimates from female muskoxen mixed-effects model, *Bulk* = β_0_ + *Animal ID* + *Year*.

| Fixed Effects | Estimate | 95% CI | t-value |
| --- | --- | --- | --- |
| Intercept | 6.13 | 5.82, 6.43 | 41.59 |
|  |  |  |  |
| Random Effects | SD | ICC |  |
| *Animal ID* | 1.50 | 0.044 |  |
| *Year* | 0.41 | 0.324 |  |
| Residual | 0.57 |  |  |
| marginal Pseudo-R^2^ = 0.000; conditional pseudo-R^2^ = 0.368 | | | |

Table SI.4b. Estimates from female muskoxen mixed-effects model, *Bulk_D_* = β_0_ + *Pregnant* + *Animal ID*.

| Fixed Effects | Estimate | 95% CI | t-value |
| --- | --- | --- | --- |
| Intercept | -3.24 | -3.47, -3.02 | -29.03 |
| *Pregnant (yes)* | 0.23 | -0.05, 0.50 | 1.68 |
|  |  |  |  |
| Random Effects | SD | ICC |  |
| *Animal ID* | 0.40 | 0.375 |  |
| Residual | 0.52 |  |  |
| marginal Pseudo-R^2^ = 0.019; conditional pseudo-R^2^ = 0.387 | | | |

Table SI.4c. Estimates from female muskoxen mixed-effects model, *Asx_D_* = β_0_ + *Age Class* + *Animal ID*.

| Fixed Effects | Estimate | 95% CI | t-value | |
| --- | --- | --- | --- | --- |
| Intercept | -0.83 | -1.29, -0.36 | -3.53 | |
| *Age Class (subadult)* | -0.38 | -0.88, 0.12 | -1.48 | |
|  |  |  |  | |
| Random Effects | SD | ICC |  | |
| *Animal ID* | 0.93 | 0.643 |  | |
| Residual | 0.69 |  |  | |
| marginal Pseudo-R^2^ = 0.016; conditional pseudo-R^2^ = 0.648 | | | |  |

Table SI.4d. Estimates from female muskoxen mixed-effects model, *Glx_D_* = β_0_ + *Animal ID*.

| Fixed Effects | Estimate | 95% CI | t-value |
| --- | --- | --- | --- |
| Intercept | -0.31 | -0.55, -0.08 | -2.72 |
|  |  |  |  |
| Random Effects | SD | ICC |  |
| *Animal ID* | 0.40 | 0.292 |  |
| Residual | 0.63 |  |  |
| marginal Pseudo-R^2^ = 0.000; conditional pseudo-R^2^ = 0.292 | | | |

Table SI.4e. Estimates from female muskoxen mixed-effects model, *Leu_D_* = β_0_ + *Diff_EM_* : *Pregnant* + *Animal ID*.

| Fixed Effects | Estimate | 95% CI | t-value |
| --- | --- | --- | --- |
| Intercept | -1.33 | -2.02, -0.64 | -3.84 |
| *Diff_EM_* | -0.01 | -0.02, -0.01 | -3.48 |
| *Pregnant (yes)* | 0.52 | 0.07, 0.96 | 2.26 |
| *Diff_EM_* : *Pregnant (yes)* | -0.02 | -0.04, 0.00 | -1.92 |
|  |  |  |  |
| Random Effects | SD | ICC |  |
| *Animal ID* | 1.39 | 0.780 |  |
| Residual | 0.74 |  |  |
| marginal Pseudo-R^2^ = 0.090; conditional pseudo-R^2^ = 0.800 | | | |

Table SI.4f. Estimates from female muskoxen mixed-effects model, *Pro_D_* = β_0_ + *Pregnant* + *Animal ID*.

| Fixed Effects | Estimate | 95% CI | t-value |
| --- | --- | --- | --- |
| Intercept | 2.49 | 1.95, 3.03 | 9.24 |
| *Pregnant (yes)* | 0.71 | 0.35, 1.07 | 3.88 |
|  |  |  |  |
| Random Effects | SD | ICC |  |
| *Animal ID* | 1.11 | 0.73 |  |
| Residual | 0.68 |  |  |
| marginal Pseudo-R^2^ = 0.045; conditional pseudo-R^2^ = 0.740 | | | |

Table SI.4g. Estimates from female muskoxen mixed-effects model, *Ser_D_* = β_0_ + *Diff_EM_* : *Age Class* + *Animal ID*.

| Fixed Effects | Estimate | 95% CI | t-value |
| --- | --- | --- | --- |
| Intercept | -1.39 | -1.70, -1.10 | -9.08 |
| *Diff_EM_* | 0.01 | 0.01, 0.02 | 3.71 |
| *Age Class (subadult)* | -0.30 | -0.89, 0.29 | -0.99 |
| *Diff_EM_* : *Age Class (subadult)* | -0.02 | -0.04, -0.01 | -2.36 |
|  |  |  |  |
| Random Effects | SD | ICC |  |
| *Animal ID* | 0.30 | 0.098 |  |
| Residual | 0.92 |  |  |
| marginal Pseudo-R^2^ = 0.143; conditional pseudo-R^2^ = 0.227 | | | |

Table SI.4h. Estimates from female muskoxen mixed-effects model, *Thr_D_* = β_0_ + *Animal ID* + *Year*.

| Fixed Effects |  | Estimate | 95% CI | t-value |
| --- | --- | --- | --- | --- |
| Intercept |  | -11.99 | -12.62, -11.34 | -37.61 |
|  |  |  |  |  |
| Random Effects |  | SD | ICC |  |
| *Animal ID* |  | 1.09 | 0.499 |  |
| *Year* |  | 0.49 | 0.101 |  |
| Residual |  | 0.98 |  |  |
| marginal Pseudo-R^2^ = 0.000; conditional pseudo-R^2^ = 0.601 | | | | |

Table SI.4i. Estimates from female muskoxen mixed-effects model, *Val_D_* = β_0_ + *Pregnant* + *Animal ID*.

| Fixed Effects | Estimate | 95% CI | t-value |
| --- | --- | --- | --- |
| Intercept | 0.58 | 0.23, 0.94 | 3.27 |
| *Pregnant (yes)* | 0.40 | -0.03, 0.81 | 1.89 |
|  |  |  |  |
| Random Effects | SD | ICC |  |
| *Animal ID* | 0.65 | 0.405 |  |
| Residual | 0.79 |  |  |
| marginal Pseudo-R^2^ = 0.023; conditional pseudo-R^2^ = 0.418 | | | |

Table SI.4j. Estimates from male muskoxen mixed-effects model, *Bulk* = β_0_ + *Animal ID*.

| Fixed Effects | Estimate | 95% CI | t-value |
| --- | --- | --- | --- |
| Intercept | 5.78 | 5.58, 5.98 | 57.78 |
|  |  |  |  |
| Random Effects | SD | ICC |  |
| *Animal ID* | 0.00 | 0.000 |  |
| Residual | 0.75 |  |  |
| marginal Pseudo-R^2^ = 0.000; conditional pseudo-R^2^ = 0.000 | | | |

Table SI.4k. Estimates from male muskoxen mixed-effects model, *Bulk_D_* = β_0_ + *Animal ID*.

| Fixed Effects | Estimate | 95% CI | t-value |
| --- | --- | --- | --- |
| Intercept | -3.45 | -3.87, -3.04 | -16.8 |
|  |  |  |  |
| Random Effects | SD | ICC |  |
| *Animal ID* | 0.68 | 0.484 |  |
| Residual | 0.70 |  |  |
| marginal Pseudo-R^2^ = 0.000; conditional pseudo-R^2^ = 0.484 | | | |

Table SI.4l. Estimates from male muskoxen mixed-effects model, *Asx_D_* = β_0_ + *Diff_EM_* + *Animal ID*.

| Fixed Effects | Estimate | 95% CI | t-value |
| --- | --- | --- | --- |
| Intercept | -1.24 | -1.88, -0.59 | -3.77 |
| *Diff_EM_* | -0.01 | -0.02, 0.00 | -2.73 |
|  |  |  |  |
| Random Effects | SD | ICC |  |
| *Animal ID* | 0.92 | 0.584 |  |
| Residual | 0.78 |  |  |
| marginal Pseudo-R^2^ = 0.090; conditional pseudo-R^2^ = 0.621 | | | |

Table SI.4m. Estimates from male muskoxen mixed-effects model, *Glx_D_* = β_0_ + *Diff_EM_* + *Animal ID*.

| Fixed Effects | Estimate | 95% CI | t-value |
| --- | --- | --- | --- |
| Intercept | -0.61 | -1.08, -0.15 | -2.59 |
| *Diff_EM_* | -0.01 | -0.01, 0.00 | -2.40 |
|  |  |  |  |
| Random Effects | SD | ICC |  |
| *Animal ID* | 0.50 | 0.312 |  |
| Residual | 0.75 |  |  |
| marginal Pseudo-R^2^ = 0.097; conditional pseudo-R^2^ = 0.378 | | | |

Table SI.4n. Estimates from male muskoxen mixed-effects model, *Leu_D_* = β_0_ + *Diff_EM_* + *Age Class* + *Animal ID*.

| Fixed Effects | Estimate | 95% CI | t-value | |
| --- | --- | --- | --- | --- |
| Intercept | -0.99 | -1.69, -0.28 | -2.87 | |
| *Diff_EM_* | -0.01 | -0.02, 0.00 | -2.91 | |
| *Age Class (subadult)* | -0.75 | -1.46, -0.05 | -2.06 | |
|  |  |  |  | |
| Random Effects | SD | ICC |  | |
| *Animal ID* | 0.63 | 0.286 |  | |
| Residual | 0.99 |  |  | |
| marginal Pseudo-R^2^ = 0.207; conditional pseudo-R^2^ = 0.434 | | | |  |

Table SI.4o. Estimates from male muskoxen mixed-effects model, *Pro_D_* = β_0_ + *Diff_EM_* : *Castrated* + *Animal ID*.

| Fixed Effects | Estimate | 95% CI | t-value |
| --- | --- | --- | --- |
| Intercept | 1.49 | 0.57, 2.40 | 3.07 |
| *Diff_EM_* | -0.02 | -0.03, 0.00 | -2.68 |
| *Castrated (yes)* | 1.38 | 0.24, 2.52 | 2.31 |
| *Diff_EM_* : *Castrated (yes)* | 0.02 | 0.00, 0.03 | 2.44 |
|  |  |  |  |
| Random Effects | SD | ICC |  |
| *Animal ID* | 0.58 | 0.291 |  |
| Residual | 0.91 |  |  |
| marginal Pseudo-R^2^ = 0.147; conditional pseudo-R^2^ = 0.396 | | | |

Table SI.4p. Estimates from male muskoxen mixed-effects model, *Ser_D_* = β_0_ + *Animal ID* + *Year*.

| Fixed Effects | Estimate | 95% CI | t-value |
| --- | --- | --- | --- |
| Intercept | -2.01 | -2.67, -1.36 | -6.16 |
|  |  |  |  |
| Random Effects | SD | ICC |  |
| *Animal ID* | 0.80 | 0.34 |  |
| *Year* | 0.64 | 0.22 |  |
| Residual | 0.91 |  |  |
| marginal Pseudo-R^2^ = 0.000; conditional pseudo-R^2^ = 0.559 | | | |

Table SI.4q. Estimates from male muskoxen mixed-effects model, *Thr_D_* = β_0_ + *Animal ID* + *Year*.

| Fixed Effects | Estimate | 95% CI | t-value |
| --- | --- | --- | --- |
| Intercept | -10.92 | -11.53, -10.30 | -36.35 |
|  |  |  |  |
| Random Effects | SD | ICC |  |
| *Animal ID* | 0.32 | 0.046 |  |
| *Year* | 0.70 | 0.219 |  |
| Residual | 1.28 |  |  |
| marginal Pseudo-R^2^ = 0.000; conditional pseudo-R^2^ = 0.265 | | | |

Table SI.4r. Estimates from male muskoxen mixed-effects model, *Val_D_* = β_0_ + *Animal ID*.

| Fixed Effects | Estimate | 95% CI | t-value |
| --- | --- | --- | --- |
| Intercept | 0.88 | 0.51, 1.25 | 4.80 |
|  |  |  |  |
| Random Effects | SD | ICC |  |
| *Animal ID* | 0.54 | 0.305 |  |
| Residual | 0.81 |  |  |
| marginal Pseudo-R^2^ = 0.000; conditional pseudo-R^2^ = 0.305 | | | |

Supplemental Information 5

Table SI.5. Model selection of female muskox with Diff_EM_ (the difference from expected mass, kg) as the response. K is number of parameters in the model, Δ AIC is the difference of AIC values from the best approximating model, and W is AICc model weight. The subscript “D” indicates that the δ^15^N value has been corrected for dietary (D) effects by subtraction of the Phe δ^15^N value.

| Random Effects Models | K | Δ AICc | W |
| --- | --- | --- | --- |
| *Animal ID* + *Year* | 4 | 0.0 | 0.82 |
| *Animal ID* | 3 | 3.0 | 0.18 |

| Fixed Effects Models Part 1  (all include random effects of *Animal ID* + *Year*) | K | Δ AICc | W |
| --- | --- | --- | --- |
| *Pregnant* | 5 | 0.0 | 0.91 |
| *Pregnant* + *Season* | 8 | 4.7 | 0.09 |
| *Season* | 7 | 17.8 | 0.00 |
| Null | 4 | 23.1 | 0.00 |

| Fixed Effects Models Part 2  (all include random effects of *Animal ID* + *Year* and additive fixed effect of *Pregnant*) | K | Δ AICc | W |
| --- | --- | --- | --- |
| *Asx_D_* + *Glx_D_* + *Leu_D_* + *Ser_D_* | 9 | 0.0 | 0.39 |
| *Asx_D_* + *Glx_D_* + *Leu_D_* + *Pro_D_* + *Ser_D_* | 10 | 1.3 | 0.20 |
| *Asx_D_* + *Glx_D_* + *Leu_D_* + *Ser_D_* + *Thr_D_* | 10 | 2.5 | 0.12 |
| *Asx_D_* + *Glx_D_* + *Leu_D_* + *Ser_D_* + *Val_D_* | 10 | 2.5 | 0.11 |
| *Asx_D_* + *Glx_D_* + *Leu_D_* + *Pro_D_* + *Ser_D_* + *Thr_D_* | 11 | 3.8 | 0.06 |
| *Asx_D_* + *Glx_D_* + *Leu_D_* + *Pro_D_* + *Ser_D_* + *Val_D_* | 11 | 3.8 | 0.06 |
| *Asx_D_* + *Glx_D_* + *Leu_D_* + *Pro_D_* + *Ser_D_* + *Val_D_* + *Thr_D_* | 12 | 6.4 | 0.02 |
| *Glx_D_* + *Leu_D_* + *Ser_D_* | 8 | 7.2 | 0.01 |
| *Glx_D_* + *Leu_D_* + *Pro_D_* + *Ser_D_* | 9 | 8.9 | 0.00 |
| *Asx_D_* + *Glx_D_* + *Ser_D_* | 8 | 9.2 | 0.00 |
| *Glx_D_* + *Leu_D_* + *Ser_D_* + *Val_D_* | 9 | 9.3 | 0.00 |
| *Glx_D_* + *Leu_D_* + *Ser_D_* + *Thr_D_* | 9 | 9.5 | 0.00 |
| *Asx_D_* + *Glx_D_* + *Pro_D_* + *Ser_D_* | 9 | 10.4 | 0.00 |
| *Glx_D_* + *Leu_D_* + *Pro_D_* + *Ser_D_* + *Val_D_* | 10 | 11.2 | 0.00 |
| *Glx_D_* + *Leu_D_* + *Pro_D_* + *Ser_D_* + *Thr_D_* | 10 | 11.2 | 0.00 |
| *Asx_D_* + *Glx_D_* + *Ser_D_* + *Val_D_* | 9 | 11.3 | 0.00 |
| *Leu_D_* + *Ser_D_* | 7 | 11.4 | 0.00 |
| *Asx_D_* + *Glx_D_* + *Ser_D_* + *Thr_D_* | 9 | 11.6 | 0.00 |
| *Asx_D_* + *Leu_D_* + *Ser_D_* | 8 | 12.6 | 0.00 |
| *Glx_D_* + *Ser_D_* | 7 | 12.9 | 0.00 |
| *Leu_D_* + *Ser_D_* + *Val_D_* | 8 | 13.3 | 0.00 |
| *Glx_D_* + *Leu_D_* + *Pro_D_* + *Ser_D_* + *Val_D_* + *Thr_D_* | 11 | 13.6 | 0.00 |
| *Leu_D_* + *Ser_D_* + *Thr_D_* | 8 | 13.6 | 0.00 |
| *Leu_D_* + *Pro_D_* + *Ser_D_* | 8 | 13.7 | 0.00 |
| *Asx_D_* + *Leu_D_* + *Ser_D_* + *Val_D_* | 9 | 13.8 | 0.00 |
| *Asx_D_* + *Glx_D_* + *Leu_D_* + *Pro_D_* | 9 | 14.5 | 0.00 |
| *Glx_D_* + *Pro_D_* + *Ser_D_* | 8 | 14.6 | 0.00 |
| *Asx_D_* + *Leu_D_* + *Pro_D_* + *Ser_D_* | 9 | 14.8 | 0.00 |
| *Asx_D_* + *Leu_D_* + *Ser_D_* + *Thr_D_* | 9 | 14.9 | 0.00 |
| *Asx_D_* + *Glx_D_* + *Leu_D_* | 8 | 15.0 | 0.00 |
| *Asx_D_* + *Leu_D_* | 7 | 15.1 | 0.00 |
| *Glx_D_* + *Ser_D_* + *Val_D_* | 8 | 15.2 | 0.00 |
| *Glx_D_* + *Ser_D_*+ *Thr_D_* | 8 | 15.2 | 0.00 |
| *Leu_D_* + *Pro_D_* + *Ser_D_* + *Val_D_* | 9 | 15.7 | 0.00 |
| *Leu_D_*+ *Pro_D_* + *Ser_D_* + *Thr_D_* | 9 | 15.9 | 0.00 |
| *Leu_D_* | 6 | 16.1 | 0.00 |
| *Asx_D_* + *Glx_D_* + *Leu_D_* + *Pro_D_* + *Thr_D_* | 10 | 16.9 | 0.00 |
| *Asx_D_* + *Glx_D_* + *Leu_D_* + *Pro_D_* + *Val_D_* | 10 | 16.9 | 0.00 |
| *Asx_D_* + *Leu_D_* + *Pro_D_* | 8 | 17.1 | 0.00 |
| *Asx_D_* + *Leu_D_* + *Val_D_* | 8 | 17.1 | 0.00 |
| *Leu_D_* + *Pro_D_* | 7 | 17.2 | 0.00 |
| *Asx_D_* + *Leu_D_* + *Thr_D_* | 8 | 17.4 | 0.00 |
| *Asx_D_* + *Glx_D_* + *Leu_D_* + *Thr_D_* | 9 | 17.4 | 0.00 |
| *Asx_D_* + *Glx_D_* + *Leu_D_* + *Val_D_* | 9 | 17.4 | 0.00 |
| *Leu_D_* + *Pro_D_* + *Ser_D_* + *Val_D_* + *Thr_D_* | 10 | 18.0 | 0.00 |
| *Leu_D_* + *Thr_D_* | 7 | 18.3 | 0.00 |
| *Leu_D_* + *Val_D_* | 7 | 18.4 | 0.00 |
| *Glx_D_* + *Leu_D_* | 7 | 18.4 | 0.00 |
| *Glx_D_* + *Leu_D_* + *Pro_D_* | 8 | 18.6 | 0.00 |
| *Asx_D_* + *Leu_D_* + *Pro_D_* + *Val_D_* | 9 | 18.9 | 0.00 |
| *Asx_D_* + *Leu_D_* + *Pro_D_* + *Thr_D_* | 9 | 19.4 | 0.00 |
| *Leu_D_* + *Pro_D_* + *Thr_D_* | 8 | 19.4 | 0.00 |
| *Asx_D_* + *Leu_D_* + *Thr_D_* + *Val_D_* | 9 | 19.4 | 0.00 |
| *Leu_D_* + *Pro_D_* + *Val_D_* | 8 | 19.6 | 0.00 |
| *Asx_D_* + *Glx_D_* + *Leu_D_* + *Thr_D_* + *Val_D_* | 10 | 19.9 | 0.00 |
| *Leu_D_* + *Thr_D_* + *Val_D_* | 8 | 20.6 | 0.00 |
| *Glx_D_* + *Leu_D_* + *Val_D_* | 8 | 20.6 | 0.00 |
| *Glx_D_* + *Leu_D_* + *Thr_D_* | 8 | 20.7 | 0.00 |
| *Glx_D_* + *Leu_D_* + *Pro_D_* + *Thr_D_* | 9 | 20.8 | 0.00 |
| *Glx_D_* + *Leu_D_* + *Pro_D_* + *Val_D_* | 9 | 20.9 | 0.00 |
| *Asx_D_* + *Glx_D_* + *Pro_D_* | 8 | 21.6 | 0.00 |
| *Asx_D_* + *Glx_D_* | 7 | 22.0 | 0.00 |
| *Ser_D_* + *Val_D_* | 7 | 22.7 | 0.00 |
| *Glx_D_* + *Leu_D_* + *Thr_D_* + *Val_D_* | 9 | 22.9 | 0.00 |
| *Asx_D_* + *Glx_D_* + *Pro_D_* + *Val_D_* | 9 | 23.4 | 0.00 |
| *Glx_D_* + *Pro_D_* | 7 | 23.4 | 0.00 |
| Null | 5 | 23.5 | 0.00 |
| *Ser_D_* | 6 | 23.9 | 0.00 |
| *Asx_D_* + *Glx_D_* + *Pro_D_* + *Thr_D_* | 9 | 24.0 | 0.00 |
| *Asx_D_* + *Glx_D_* + *Val_D_* | 8 | 24.1 | 0.00 |
| *Asx_D_* + *Glx_D_* + *Thr_D_* | 8 | 24.4 | 0.00 |
| *Val_D_* | 6 | 24.4 | 0.00 |
| *Asx_D_* + *Ser_D_* + *Val_D_* | 8 | 24.7 | 0.00 |
| *Pro_D_* + *Ser_D_* + *Val_D_* | 8 | 25.0 | 0.00 |
| *Ser_D_* + *Thr_D_* + *Val_D_* | 8 | 25.0 | 0.00 |
| *Glx_D_* + *Val_D_* | 7 | 25.5 | 0.00 |
| *Glx_D_* + *Thr_D_* | 7 | 25.5 | 0.00 |
| *Asx_D_* + *Val_D_* | 7 | 25.6 | 0.00 |
| *Asx_D_* | 6 | 25.7 | 0.00 |
| *Glx_D_* + *Pro_D_* + *Val_D_* | 8 | 25.7 | 0.00 |
| *Bulk* | 6 | 25.7 | 0.00 |
| *Bulk_D_* | 6 | 25.7 | 0.00 |
| *Pro_D_* | 6 | 25.7 | 0.00 |
| *Thr_D_* | 6 | 25.7 | 0.00 |
| *Glx_D_* + *Pro_D_* + *Thr_D_* | 8 | 25.8 | 0.00 |
| *Pro_D_* + *Ser_D_* | 7 | 25.9 | 0.00 |
| *Asx_D_* + *Ser_D_* | 7 | 26.2 | 0.00 |
| *Pro_D_* + *Val_D_* | 7 | 26.2 | 0.00 |
| *Ser_D_* + *Thr_D_* | 7 | 26.2 | 0.00 |
| *Asx_D_* + *Glx_D_* + *Thr_D_* + *Val_D_* | 9 | 26.5 | 0.00 |
| *Thr_D_* + *Val_D_* | 7 | 26.7 | 0.00 |
| *Asx_D_* + *Pro_D_* + *Ser_D_* + *Val_D_* | 9 | 27.1 | 0.00 |
| *Asx_D_* + *Ser_D_* + *Thr_D_* + *Val_D_* | 9 | 27.1 | 0.00 |
| *Pro_D_* + *Ser_D_* + *Val_D_* + *Thr_D_* | 9 | 27.4 | 0.00 |
| *Asx_D_* + *Pro_D_* + *Val_D_* | 8 | 27.7 | 0.00 |
| *Glx_D_* + *Thr_D_* + *Val_D_* | 8 | 27.9 | 0.00 |
| *Asx_D_* + *Pro_D_* | 7 | 27.9 | 0.00 |
| *Asx_D_* + *Thr_D_* | 7 | 28.0 | 0.00 |
| *Asx_D_* + *Thr_D_* + *Val_D_* | 8 | 28.0 | 0.00 |
| *Pro_D_* + *Thr_D_* | 7 | 28.0 | 0.00 |
| *Asx_D_* + *Pro_D_* + *Ser_D_* | 8 | 28.2 | 0.00 |
| *Pro_D_* + *Ser_D_* + *Thr_D_* | 8 | 28.2 | 0.00 |
| *Asx_D_* + *Ser_D_* + *Thr_D_* | 8 | 28.5 | 0.00 |
| *Pro_D_* + *Thr_D_* + *Val_D_* | 8 | 28.6 | 0.00 |
| *Asx_D_* + *Pro_D_* + *Thr_D_* + *Val_D_* | 9 | 30.1 | 0.00 |

| Models  (all include random effects of *Animal ID* + *Year*; *P* = *Pregnant*) | K | Δ AICc | W |
| --- | --- | --- | --- |
| *P* + *Asx_D_* + *Glx_D_* + *Leu_D_* + *Ser_D_* | 9 | 0.0 | 0.27 |
| *P* + *Asx_D_* + *Glx_D_* + *Leu_D_* + *Ser_D_* + *Asx_D_* : *P* | 10 | 1.5 | 0.13 |
| *P* + *Leu_D_* + *Asx_D_* + *Glx_D_* + *Ser_D_* + *Leu_D_* : *P* | 10 | 2.1 | 0.09 |
| *P* + *Asx_D_* + *Glx_D_* + *Leu_D_* + *Ser_D_* + *Ser_D_* : *P* | 10 | 2.3 | 0.09 |
| *P* + *Asx_D_* + *Glx_D_* + *Leu_D_* + *Ser_D_* + *Asx_D_* : *P* + *Leu_D_* : *P* | 11 | 2.3 | 0.09 |
| *P* + *Asx_D_* + *Glx_D_* + *Leu_D_* + *Ser_D_* + *Glx_D_* : *P* | 10 | 2.5 | 0.08 |
| *P* + *Asx_D_* + *Glx_D_* + *Leu_D_* + *Ser_D_* + *Asx_D_* : *P* + *Glx_D_* : *P* | 11 | 3.3 | 0.05 |
| *P* + *Asx_D_* + *Glx_D_* + *Leu_D_* + *Ser_D_* + *Asx_D_* : *P* + *Ser_D_* : *P* | 11 | 4.0 | 0.04 |
| *P* + *Asx_D_* + *Glx_D_* + *Leu_D_* + *Ser_D_* + *Leu_D_* : *P* + *Ser_D_* : *P* | 11 | 4.2 | 0.03 |
| *P* + *Asx_D_* + *Glx_D_* + *Leu_D_* + *Ser_D_* + *Glx_D_* : *P* + *Ser_D_* : *P* | 11 | 4.3 | 0.03 |
| *P* + *Asx_D_* + *Glx_D_* + *Leu_D_* + *Ser_D_* + *Glx_D_* : *P* + *Leu_D_* : *P* | 11 | 4.5 | 0.03 |
| *P* + *Asx_D_* + *Glx_D_* + *Leu_D_* + *Ser_D_* + *Asx_D_* : *P* + *Glx_D_* : *P* + *Leu_D_* : *P* | 12 | 4.7 | 0.03 |
| *P* + *Asx_D_* + *Glx_D_* + *Leu_D_* + *Ser_D_* + *Asx_D_* : *P* + *Leu_D_* : *P* + *Ser_D_* : *P* | 12 | 4.8 | 0.02 |
| *P* + *Asx_D_* + *Glx_D_* + *Leu_D_* + *Ser_D_* + *Asx_D_* : *P* + *Glx_D_* : *P* + *Ser_D_* : *P* | 12 | 4.9 | 0.02 |
| *P* + *Asx_D_* + *Glx_D_* + *Leu_D_* + *Ser_D_* + *Glx_D_* : *P* + *Leu_D_* : *P* + *Ser_D_* : *P* | 12 | 6.7 | 0.01 |
| *P* + *Asx_D_* + *Glx_D_* + *Leu_D_* + *Ser_D_* + *Asx_D_* : *P* + *Glx_D_* : *P* + *Leu_D_* : *P* + *Ser_D_* : *P* | 13 | 6.9 | 0.01 |

Supplemental Information 6

Table SI.6. Model selection of male muskox with Diff_EM_ (the difference from expected mass, kg) as the response. K is number of parameters in the model, Δ AIC is the difference of AIC values from the best approximating model, and W is AICc model weight. The subscript “D” indicates that the δ^15^N value has been corrected for dietary (D) effects by subtraction of the Phe δ^15^N value.

| Random Effects Models | K | Δ AICc | W |
| --- | --- | --- | --- |
| *Animal ID* + *Year* | 4 | 0.0 | 1.00 |
| *Animal ID* | 3 | 15.3 | 0.00 |

| Fixed Effects Models Part 1  (all include random effects of *Animal ID* and *Year*) | K | Δ AICc | W |
| --- | --- | --- | --- |
| Null | 4 | 0.0 | 0.66 |
| *Castrated* | 5 | 1.6 | 0.29 |
| *Season* | 7 | 6.1 | 0.03 |
| *Castrated* + *Season* | 8 | 7.8 | 0.01 |

| Fixed Effects Models Part 2  (all include random effects of *Animal ID* + *Year*) | K | Δ AICc | W |
| --- | --- | --- | --- |
| *Glx_D_* + *Leu_D_* + *Pro_D_* | 7 | 0.0 | 0.17 |
| *Leu_D_* | 5 | 1.5 | 0.08 |
| *Asx_D_* + *Glx_D_* + *Leu_D_* + *Pro_D_* | 8 | 2.5 | 0.05 |
| *Glx_D_* + *Leu_D_* + *Pro_D_* + *Val_D_* | 8 | 2.5 | 0.05 |
| *Glx_D_* + *Leu_D_* + *Pro_D_* + *Ser_D_* | 8 | 2.7 | 0.04 |
| *Glx_D_* + *Leu_D_* + *Pro_D_* + *Thr_D_* | 8 | 2.7 | 0.04 |
| *Glx_D_* + *Leu_D_* | 6 | 3.1 | 0.04 |
| *Leu_D_* + *Pro_D_* | 6 | 3.2 | 0.03 |
| *Leu_D_* + *Ser_D_* | 6 | 3.4 | 0.03 |
| *Asx_D_* + *Leu_D_* + *Pro_D_* | 7 | 3.9 | 0.02 |
| *Asx_D_* + *Leu_D_* | 6 | 3.9 | 0.02 |
| *Leu_D_* + *Val_D_* | 6 | 4.0 | 0.02 |
| *Leu_D_* + *Thr_D_* | 6 | 4.1 | 0.02 |
| *Leu_D_* + *Pro_D_* + *Ser_D_* | 7 | 4.1 | 0.02 |
| *Asx_D_* + *Glx_D_* + *Leu_D_* | 7 | 4.8 | 0.02 |
| *Glx_D_* + *Leu_D_* + *Val_D_* | 7 | 5.1 | 0.01 |
| *Asx_D_* + *Glx_D_* + *Leu_D_* + *Pro_D_* + *Val_D_* | 9 | 5.2 | 0.01 |
| *Asx_D_* + *Glx_D_* + *Leu_D_* + *Pro_D_* + *Thr_D_* | 9 | 5.3 | 0.01 |
| *Asx_D_* + *Glx_D_* + *Leu_D_* + *Pro_D_* + *Ser_D_* | 9 | 5.3 | 0.01 |
| *Glx_D_* + *Leu_D_* + *Pro_D_* + *Ser_D_* + *Val_D_* | 9 | 5.3 | 0.01 |
| *Glx_D_* + *Leu_D_* + *Pro_D_* + *Ser_D_* + *Thr_D_* | 9 | 5.6 | 0.01 |
| *Glx_D_* + *Leu_D_* + *Ser_D_* | 7 | 5.6 | 0.01 |
| *Glx_D_* + *Leu_D_* + *Thr_D_* | 7 | 5.7 | 0.01 |
| *Asx_D_* + *Leu_D_* + *Pro_D_* + *Ser_D_* | 8 | 5.7 | 0.01 |
| *Leu_D_* + *Pro_D_* + *Thr_D_* | 7 | 5.8 | 0.01 |
| *Leu_D_* + *Pro_D_* + *Val_D_* | 7 | 5.8 | 0.01 |
| *Leu_D_* + *Ser_D_* + *Val_D_* | 7 | 5.8 | 0.01 |
| Null | 4 | 5.8 | 0.01 |
| *Glx_D_* + *Pro_D_* | 6 | 6.0 | 0.01 |
| *Leu_D_* + *Ser_D_* + *Thr_D_* | 7 | 6.0 | 0.01 |
| *Asx_D_* + *Leu_D_* + *Ser_D_* | 7 | 6.0 | 0.01 |
| *Asx_D_* | 5 | 6.3 | 0.01 |
| *Asx_D_* + *Leu_D_* + *Val_D_* | 7 | 6.4 | 0.01 |
| *Asx_D_* + *Leu_D_* + *Thr_D_* | 7 | 6.6 | 0.01 |
| *Leu_D_* + *Thr_D_* + *Val_D_* | 7 | 6.6 | 0.01 |
| *Asx_D_* + *Leu_D_* + *Pro_D_* + *Val_D_* | 8 | 6.6 | 0.01 |
| *Asx_D_* + *Leu_D_* + *Pro_D_* + *Thr_D_* | 8 | 6.7 | 0.01 |
| *Glx_D_* + *Val_D_* | 6 | 6.8 | 0.01 |
| *Leu_D_* + *Pro_D_* + *Ser_D_* + *Val_D_* | 8 | 6.8 | 0.01 |
| *Leu_D_* + *Pro_D_* + *Ser_D_* + *Thr_D_* | 8 | 6.8 | 0.01 |
| *Glx_D_* + *Pro_D_* + *Ser_D_* | 7 | 7.1 | 0.00 |
| *Asx_D_* + *Glx_D_* + *Leu_D_* + *Val_D_* | 8 | 7.1 | 0.00 |
| *Glx_D_* + *Ser_D_* | 6 | 7.2 | 0.00 |
| *Bulk* | 5 | 7.4 | 0.00 |
| *Asx_D_* + *Glx_D_* + *Leu_D_* + *Thr_D_* | 8 | 7.5 | 0.00 |
| *Asx_D_* + *Glx_D_* + *Leu_D_* + *Ser_D_* | 8 | 7.5 | 0.00 |
| *Glx_D_* + *Leu_D_* + *Thr_D_* + *Val_D_* | 8 | 7.7 | 0.00 |
| *Asx_D_* + *Pro_D_* | 6 | 7.7 | 0.00 |
| *Glx_D_* + *Leu_D_* + *Ser_D_* + *Val_D_* | 8 | 7.7 | 0.00 |
| *Glx_D_* + *Pro_D_* + *Val_D_* | 7 | 7.7 | 0.00 |
| *Asx_D_* + *Glx_D_* | 6 | 7.9 | 0.00 |
| *Glx_D_* + *Thr_D_* | 6 | 7.9 | 0.00 |
| *Asx_D_* + *Val_D_* | 6 | 8.0 | 0.00 |
| *Pro_D_* | 5 | 8.1 | 0.00 |
| *Asx_D_* + *Glx_D_* + *Leu_D_* + *Pro_D_* + *Ser_D_* + *Val_D_* | 10 | 8.1 | 0.00 |
| *Glx_D_* + *Leu_D_* + *Pro_D_* + *Ser_D_* + *Val_D_* + *Thr_D_* | 10 | 8.1 | 0.00 |
| *Bulk_D_* | 5 | 8.2 | 0.00 |
| *Ser_D_* | 5 | 8.2 | 0.00 |
| *Val_D_* | 5 | 8.2 | 0.00 |
| *Thr_D_* | 5 | 8.2 | 0.00 |
| *Asx_D_* + *Glx_D_* + *Leu_D_* + *Pro_D_* + *Ser_D_* + *Thr_D_* | 10 | 8.3 | 0.00 |
| *Glx_D_* + *Leu_D_* + *Ser_D_* + *Thr_D_* | 8 | 8.4 | 0.00 |
| *Asx_D_* + *Leu_D_* + *Ser_D_* + *Val_D_* | 8 | 8.5 | 0.00 |
| *Asx_D_* + *Glx_D_* + *Pro_D_* | 7 | 8.5 | 0.00 |
| *Glx_D_* + *Pro_D_* + *Thr_D_* | 7 | 8.6 | 0.00 |
| *Asx_D_* + *Ser_D_* | 6 | 8.7 | 0.00 |
| *Asx_D_* + *Leu_D_* + *Ser_D_* + *Thr_D_* | 8 | 8.7 | 0.00 |
| *Asx_D_* + *Thr_D_* | 6 | 8.8 | 0.00 |
| *Glx_D_* + *Ser_D_* + *Val_D_* | 7 | 8.9 | 0.00 |
| *Asx_D_* + *Leu_D_* + *Thr_D_* + *Val_D_* | 8 | 9.1 | 0.00 |
| *Glx_D_* + *Thr_D_* + *Val_D_* | 7 | 9.2 | 0.00 |
| *Asx_D_* + *Glx_D_* + *Val_D_* | 7 | 9.4 | 0.00 |
| *Leu_D_* + *Pro_D_* + *Ser_D_* + *Val_D_* + *Thr_D_* | 9 | 9.6 | 0.00 |
| *Asx_D_* + *Glx_D_* + *Leu_D_* + *Thr_D_* + *Val_D_* | 9 | 9.7 | 0.00 |
| *Asx_D_* + *Glx_D_* + *Ser_D_* | 7 | 9.7 | 0.00 |
| *Asx_D_* + *Pro_D_* + *Val_D_* | 7 | 9.7 | 0.00 |
| *Glx_D_* + *Ser_D_* + *Thr_D_* | 7 | 9.8 | 0.00 |
| *Asx_D_* + *Glx_D_* + *Pro_D_* + *Ser_D_* | 8 | 9.8 | 0.00 |
| *Asx_D_* + *Glx_D_* + *Leu_D_* + *Ser_D_* + *Val_D_* | 9 | 9.9 | 0.00 |
| *Asx_D_* + *Pro_D_* + *Ser_D_* | 7 | 10.2 | 0.00 |
| *Asx_D_* + *Pro_D_* + *Thr_D_* | 7 | 10.2 | 0.00 |
| *Asx_D_* + *Glx_D_* + *Pro_D_* + *Val_D_* | 8 | 10.3 | 0.00 |
| *Asx_D_* + *Glx_D_* + *Leu_D_* + *Ser_D_* + *Thr_D_* | 9 | 10.3 | 0.00 |
| *Pro_D_* + *Val_D_* | 6 | 10.5 | 0.00 |
| *Asx_D_* + *Glx_D_* + *Thr_D_* | 7 | 10.5 | 0.00 |
| *Ser_D_* + *Val_D_* | 6 | 10.6 | 0.00 |
| *Pro_D_* + *Ser_D_* | 6 | 10.6 | 0.00 |
| *Pro_D_* + *Thr_D_* | 6 | 10.6 | 0.00 |
| *Thr_D_* + *Val_D_* | 6 | 10.6 | 0.00 |
| *Asx_D_* + *Ser_D_* + *Val_D_* | 7 | 10.6 | 0.00 |
| *Asx_D_* + *Thr_D_* + *Val_D_* | 7 | 10.6 | 0.00 |
| *Ser_D_* + *Thr_D_* | 6 | 10.7 | 0.00 |
| *Asx_D_* + *Glx_D_* + *Leu_D_* + *Pro_D_* + *Ser_D_* + *Val_D_* + *Thr_D_* | 11 | 11.0 | 0.00 |
| *Asx_D_* + *Glx_D_* + *Pro_D_* + *Thr_D_* | 8 | 11.2 | 0.00 |
| *Asx_D_* + *Ser_D_* + *Thr_D_* | 7 | 11.3 | 0.00 |
| *Asx_D_* + *Glx_D_* + *Ser_D_* + *Val_D_* | 8 | 11.6 | 0.00 |
| *Asx_D_* + *Glx_D_* + *Thr_D_* + *Val_D_* | 8 | 11.9 | 0.00 |
| *Asx_D_* + *Glx_D_* + *Ser_D_* + *Thr_D_* | 8 | 12.4 | 0.00 |
| *Asx_D_* + *Pro_D_* + *Ser_D_* + *Val_D_* | 8 | 12.4 | 0.00 |
| *Asx_D_* + *Pro_D_* + *Thr_D_* + *Val_D_* | 8 | 12.5 | 0.00 |
| *Asx_D_* + *Pro_D_* + *Ser_D_* + *Thr_D_* | 8 | 12.9 | 0.00 |
| *Pro_D_* + *Ser_D_* + *Val_D_* | 7 | 13.0 | 0.00 |
| *Pro_D_* + *Thr_D_* + *Val_D_* | 7 | 13.0 | 0.00 |
| *Ser_D_* + *Thr_D_* + *Val_D_* | 7 | 13.2 | 0.00 |
| *Pro_D_* + *Ser_D_* + *Thr_D_* | 7 | 13.2 | 0.00 |
